# Information about the natural history of acute infections commonly seen in primary care: a systematic review of clinical practice guidelines

**DOI:** 10.1186/s12879-022-07887-1

**Published:** 2022-12-01

**Authors:** Kwame Peprah Boaitey, Mina Bakhit, Natalia Krzyzaniak, Tammy C. Hoffmann

**Affiliations:** Faculty of Health Sciences and Medicine, Institute for Evidence-Based Healthcare, Bond University, 14 University Dr, Robina, QLD 4229 Australia

**Keywords:** Natural history, Primary care, Acute infections, Clinical practice guidelines, Antibiotic stewardship

## Abstract

**Background:**

Many of the acute infections that are seen in primary care and sometimes managed with antibiotics are self-resolving and antibiotics may be unnecessary. Information about the natural history of these infections underpins antibiotic stewardship strategies such as delayed prescribing and shared decision making, yet whether it’s reported in guidelines is unknown. We examined, in clinical guidelines, the reporting of natural history information and relevant antibiotic stewardship strategies for acute infections commonly seen in primary care.

**Methods:**

A systematic review of national and international guidelines (2010 onwards), available electronically, for managing acute infections (respiratory, urinary, or skin and soft tissue). We searched MEDLINE, CINAHL, EMBASE, TRIP, and GIN databases and websites of 22 guideline-publishing organisations.

**Results:**

We identified 82 guidelines, covering 114 eligible infections**.** Natural history information was reported in 49 (59.8%) of the guidelines and 66 (57.9%) of the reported conditions, most commonly for respiratory tract infections. Quantitative information about the expected infection duration was provided for 63.5% (n = 42) of the infections. Delayed antibiotic prescribing strategy was recommended for 34.2% (n = 39) of them and shared decision making for 21% (n = 24).

**Conclusions:**

Just over half of the guidelines for acute infections that are commonly managed in primary care and sometimes with antibiotics contained natural history information. As many of these infections spontaneously improve, this is a missed opportunity to disseminate this information to clinicians, promote antibiotic stewardship, and facilitate conversations with patients and informed decision making.

*Systematic review registration* CRD42021247048

**Supplementary Information:**

The online version contains supplementary material available at 10.1186/s12879-022-07887-1.

## Introduction

Antibiotic resistance is a major public health concern that threatens the effective treatment of bacterial infections [1]. Overuse and misuse of antibiotics are major drivers of antibiotic resistance [2]. Optimising the use of existing antibiotics has been identified as a major strategy in preventing resistance to antibiotics [3]. Between 52% and 80% of antibiotic prescribing is estimated to arise from primary care consultations, with some of this prescribing for self-limiting acute infections and hence unnecessary [4–7].

Acute infections are one of the common reasons for patients to attend primary care [8], with many infections needing symptom management, but not necessarily antibiotics [9, 10] The natural history of these conditions (defined as the course of a disease process over time, in the absence of treatment) [11] should be considered as part of clinical decision-making. Doing so may help to reduce antibiotic overuse.

Clinical practice guidelines are systematically developed statements to assist decision-making about appropriate health care for specific clinical circumstances [12]. They are tools to convey evidence-based information to clinicians with the goal of improving care quality and health outcomes [12, 13]. Given the high rate of antibiotic prescribing in primary care, various antibiotic stewardship clinical strategies are recommended, including near-patient testing, delayed antibiotic prescribing (or “wait and see”) and shared decision making [14–17]. Some of these strategies require natural history information [18, 19] and it is unknown if guidelines for common acute infections provide this information.

The inclusion of natural history information in guidelines may help to facilitate conversations between clinicians and patients about the options for managing acute infections that are sometimes treated with antibiotics [20, 21]. Many patients and clinicians have unrealistic expectations about the effectiveness of antibiotics and are unaware that not using antibiotics is sometimes a legitimate management option [22–24]. Discussion about the options of using and not using antibiotics, along with the benefits and harms of each option, is vital for facilitating shared and informed decision-making about managing the infection [25, 26]. Providing clinicians with natural history information may also assist them to recognise subgroups of patients who may benefit from immediate antibiotic prescribing [9, 27]. The aims of this study were to examine the reporting of natural history information in guidelines about acute infections that are commonly seen in primary care and sometimes managed with antibiotics and whether references supporting the information were provided by research. We also assessed the reporting of relevant antibiotic stewardship clinical strategies that utilise natural history information (shared decision making anddelayed antibiotic prescribing).

## Methods

### Design

This systematic review follows the Preferred Reporting Items for Systematic Review and Meta-Analysis (PRISMA 2020) reporting guideline [28] and the systematic review of clinical practice guidelines methodological guide by Johnston et al. 2019 [29]. We registered the protocol in PROSPERO- CRD42021247048 [30].

### Data sources and searches

The search strategy was developed with the input of an experienced information specialist. Multiple databases were searched from 2010 to February 2021: MEDLINE (Ovid), CINAHL, and EMBASE.. We supplemented our databases search by manually searching the Turning Research into Practice (TRIP) medical database (which uses multiple strategies to locate guidelines) using a combination of MESH heading and free-text words, the Guideline International Network (GIN) and the websites of 22 guideline development organisations (see Additional file 1: A1 and A2 for the complete search strategy and the list of guideline publishing institution databases searched).

### Eligibility criteria

Guideline eligibility criteria were: published after 2010, electronically available with access to full text, produced by an international or national organisation involved in the publication of guidelines, and contained clinical recommendations on the management of acute infections that are commonly seen in primary care and may be managed with antibiotics (including acute respiratory infections (ARIs), lower urinary tract infections (UTIs), skin and soft tissue infections (SSTIs), conjunctivitis). We selected these conditions because they are among the most common infections that are managed in primary care [8, 31, 32]. If several versions of a guideline by the same publishing authority were available, we included only the most recent version. Supplementary materials were considered as an extension of each guideline. No language limitations were imposed. For non-English language guidelines, we used Google Translate to determine their eligibility and extracted the relevant sections of the guideline after translating them to English.

We excluded consensus statements and expert group advice with no specific recommendations on managing the eligible conditions. Guidelines published in other forms such as books, booklets, government documents not issued as guidelines, workshop reports, and operational manuals, were excluded, as were those targeting settings other than primary care (e.g., hospitals). We excluded acute otitis media guidelines targeted at children younger than 2 years as these patients are typically recommended to receive antibiotics. Guidelines were eligible regardless of their recommendations on whether antibiotics should or/not be prescribed (see Additional file 1: B for inclusion and exclusion criteria).

### Guideline selection process

Two reviewers (KP and MB) independently screened the titles and abstracts and then checked the full text of relevant guidelines to assess eligibility. Any disagreement was resolved by consensus or discussion with a third author (TH).

### Data extraction process

We extracted the data from each guideline using a customised spreadsheet which was piloted by three authors (KB, NK, and MB) using a random sample of 10 eligible guidelines and minor modifications subsequently made. Each guideline was assigned a unique code to facilitate data extraction and analysis. A pair of authors (KP, and MB or NK) independently extracted the data outlined in Box 1 1. We extracted natural history information from the text, tables, and additional documents wherever mentioned in the guideline. We defined natural history as the course of an infection over time, without treatment (in this case, antibiotics).

Box 1: characteristics and outcomes for which data were extracted

1. **Guideline characteristics:** title, publisher/authors, link to the source, year of publication, version, country/ region of the published guideline, language, intended users.
2. **Condition’s characteristics:** targeted body system, type of condition, targeted age group, relevant diagnostic criteria
3. **Outcome data:**
4. 3a. Natural history: information present or not for each eligible condition, citing references details (year of publication, study type), verbatim wording of the natural history information (including duration of the illness if untreated)
5. 3b. Antibiotic stewardship strategies: delayed prescribing or shared decision making information present or not, verbatim wording of delayed prescribing/shared decision making information, mention of patient decision aids with a link to and/or the location of any relevant resources/materials mentioned.

### Assessment of the quality of guidelines

A pair of authors (KP, and NK or MB) used the Appraisal of Guidelines for Research and Evaluation (AGREE II) assessment tool [33] to assess the methodological rigour, transparency, quality, and reporting of each guideline. Guidelines were evaluated using all 23 items, grouped into six domains: scope and purpose, stakeholder involvement, rigour of development, clarity of presentation, applicability, and editorial independence. Each item was scored on a Likert scale of seven points (where 1 = strongly disagree and 7 = strongly agree). To achieve rating consistency, assessors completed the training activity on the AGREE II website, independently assessed five eligible guidelines, and discussed their ratings. Disagreements were resolved by consensus discussion and when not obtained, by discussion with another author (TH).

### Data synthesis and analysis

We calculated descriptive statistics using Microsoft^®^ Excel^®^ 365 (Microsoft, Redmond, WA, USA). We categorised natural history reporting into “extended” or “basic” [34], with reporting classified as “extended” if it included quantitative information about the likely duration of the infection or as “basic” if the guideline mentioned that the condition might spontaneously improve but provided no quantitative information. We grouped conjunctivitis with respiratory tract infections. As per the AGREE II recommendations, each domain score was calculated by summing up all the scores of the individual items in each domain and then standardising as follows: (score obtained − minimum possible score) / (maximum possible score − minimum possible score) to get a standardised score. We presented the individual score of each domain as a percentage.

### Modifications from the protocol

We clarified that the inclusion criteria were to include only guidelines published after 2010 to ensure currency in the sample of included guidelines, as most are updated within at least 10 years of publication. We also clarified the search database to include the GIN database. We did not describe in the protocol that verbatim information would be categorised as basic or extended.

## Results

**Fig. 1:**
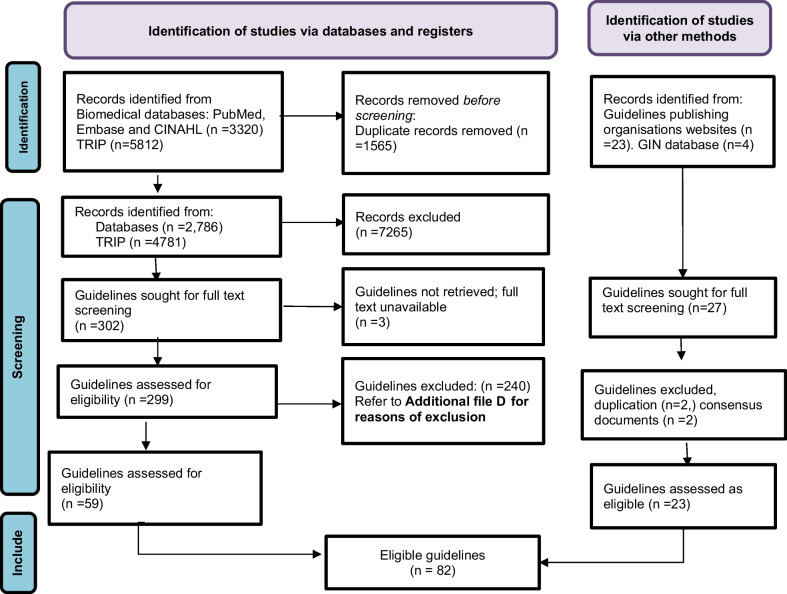
PRISMA 2020 flow diagram Adapted from: Page MJ, McKenzie JE, Bossuyt PM, Boutron I, Hoffmann TC, Mulrow CD, et al. The PRISMA 2020 statement: an updated guideline for reporting systematic reviews. BMJ 2021;372:n71. https://doi.org/10.1136/bmj.n71

A total of 9132 records were identified from the biomedical and TRIP databases. After removal of duplicates and title and abstract screening, 302 full texts were checked, with 59 guidelines assessed as eligible. An additional search of the GIN database and websites of 22 guideline development or publishing organisations yielded 23 guidelines (see Fig. 1 for PRISMA flow diagram). Eighty-two guidelines (see list in Additional file 1: C for the complete list of included guidelines), covering 114 eligible acute infections, were included.

### Characteristics of included guidelines

Table 1 shows the characteristics of the included guidelines. Of the 82 guidelines, 5 were from multi-country organisations and professional societies, with the remainder from 19 countries. Half (50%, n = 41) were developed in Europe and 23.2% (n = 19) in the United States of America (USA), with 42.7% (n = 35) published between 2017 and 2019. More than half (61%, n = 50) focused on managing acute respiratory infections. Only 16% (n = 13) of guidelines scored ≥ 70% across all domains of the AGREE II. Most scored higher in the domains of scope and purpose, and clarity of presentation, with lower scores for applicability and editorial independence. See Additional file 1: E for the complete details about the AGREE II appraisal scores of included guidelines.

**Table 1:** Characteristics of included guidelines (n = 82)

Characteristic or classification	n (%)
Year of publication	
2011–2013	15 (18.3)
2014–2016	21 (25.6)
2017–2019	35 (42.7)
2020-present	11 (13.4)
Continent of origin	
Europe	41 (50.0)
North America	19 (23.2)
Asia	12 (14.6)
Australia	3 (3.7)
South America	2 (2.4)
Other*	5 (6.1)
Language of publication	
English	57 (69.5)
Dutch	7 (8.5)
Finnish	5 (6.1)
German	4 (4.9)
Other**	9 (11.0)
Body system addressed by guideline	
Respiratory***	50 (61.0)
Urinary	20 (24.4)
Skin and soft tissue (SSTI)	12 (14.6)

AGREE II domain scores across all guidelines	
Domain score	Median score (range%)
Scope and purpose	86.1 (38.9–100)
Clarity of presentation	84.8 (50.0–100)
Rigour and development	79.2 (11.5–100)
Stakeholder involvement	77.8 (16.7–100)
Editorial independence	70.8. (4.2–100)
Applicability	64.5 (20.8–91.7)

*Multi-country organizations published guidelines

**Other languages included French, Korean, Spanish, and Russian

***Including conjunctivitis

### The inclusion of natural history information for acute infections in the guidelines

Of the 82 guidelines, 49 (59.8%) mentioned natural history information for at least one of the eligible infections. Of the 114 infections covered across the guidelines, natural history information was reported in 66 (57.9%), most commonly for respiratory tract infections (Table 2).

**Table 2:** Number and percentage of eligible conditions for which natural history information and references were provided

	Respiratory (n = 68)	Urinary (n = 26)	SSTI (n = 20)	Total (n = 114)
Natural history information reported				
Yes, n (%)	53 (78.0)	10 (38.5)	3 (15.0)	66 (57.9)
*Supporting reference for the information provided				
Yes, n (%)	49 (92.5)	9 (90)	2 (66.7)	60 (91)

*For conditions with reported natural history information

**Fig. 2:**
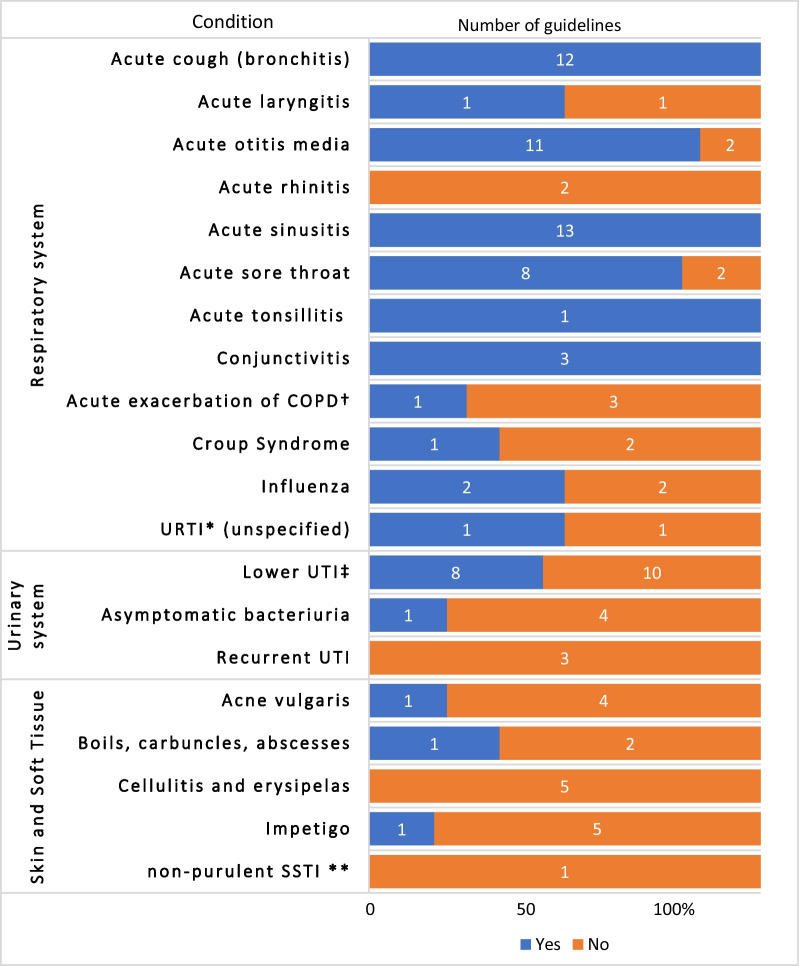
Percentage of guidelines that, for each of the acute infections, reported natural history information (number of guidelines addressing each infection shown within each bar)

Natural history information was provided in all guidelines that addressed acute bronchitis, acute sinusitis, tonsilitis, and conjunctivitis. For other infections, such as acute rhinitis, recurrent UTI, and cellulitis, it was not reported in any of the guidelines (see Fig. 2).

### The reporting of natural history information

**Fig. 3:**
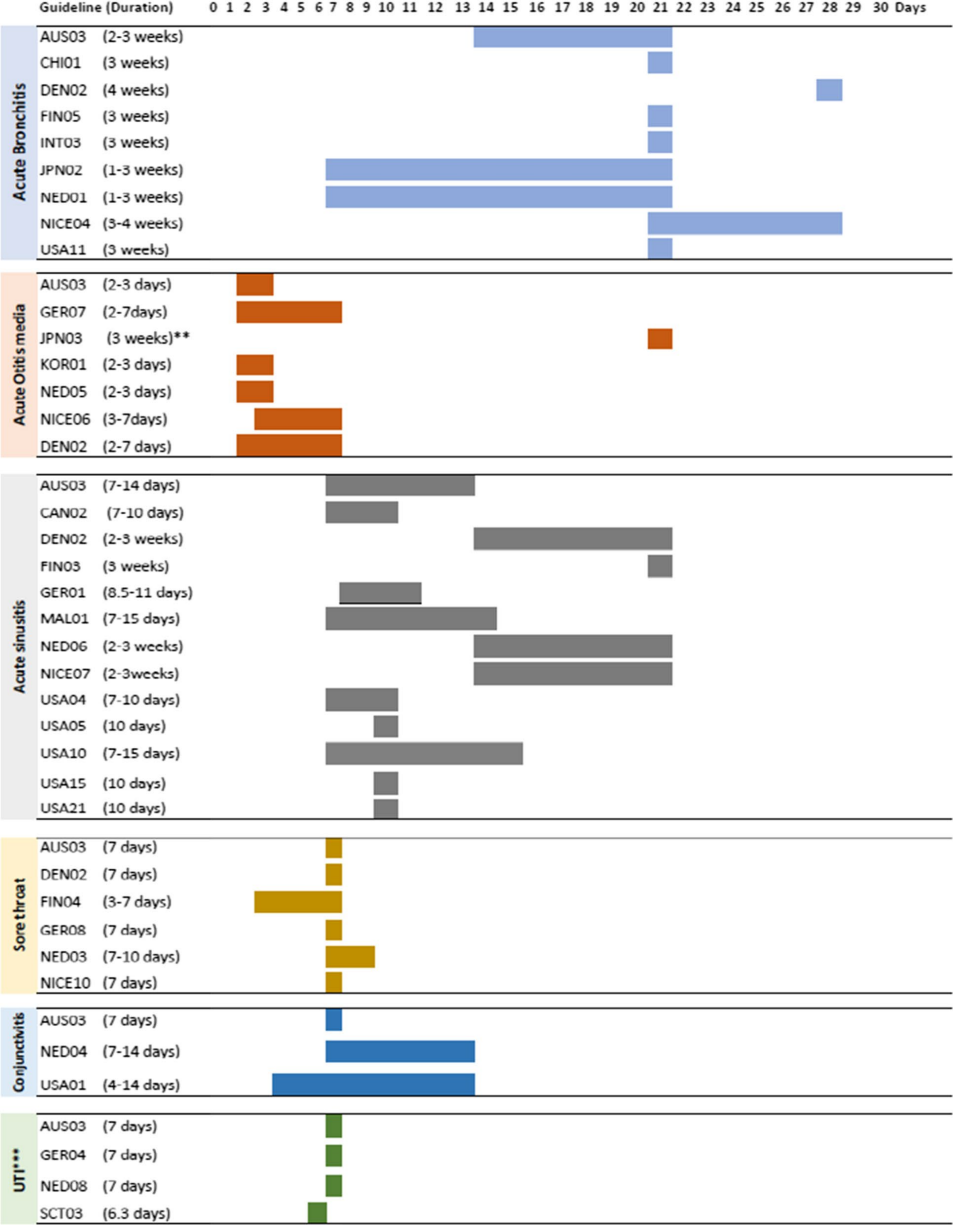
The reported duration of infections across guidelines*. *Only conditions that are reported in two or more guidelines. **Duration reported as part of the definition for the acute condition adopted by the guideline. ***Urinary tract infections

Table 3 contains verbatim examples of how guidelines reported natural history information. Of the pieces of natural history information in the guidelines, 63.6% (n = 42) were reported at an extended level. There was variation in the estimated duration of symptoms across guidelines addressing the same condition. For example, an Australian guideline (AUS 03) reported the duration for acute sinusitis to be 7–14 days, while a United Kingdom (UK) guideline (NICE 07) reported 14–21 days. Some guidelines provided a range for the expected infection duration, while others mentioned the estimated number of days those symptoms will last. Figure 3 illustrates reporting of the quantitative duration of natural history for infections that are reported in two or more guidelines. Additional file 1: F contains a list of duration for infections reported by only one guideline.

**Table 3:** Verbatim examples of natural history reporting for acute infections in guidelines, grouped by level of description and body system

System	Extended level*	Basic level**
Respiratory	*Examples* *Acute Bronchitis*	
	AUS 03: Acute bronchitis is a self-limiting lower respiratory tract infection. Explain that the cough lasts on average for 2 to 3 weeks and 90% of patients have cough resolution by four weeks. Occasionally, the cough may persist for up to 8 weeks	DEN02: In uncomplicated acute bronchitis in otherwise healthy individuals, no marginal effect of antibiotic treatment measured on the duration or symptom reduction
	*Acute sinusitis*	
	NICE07: Acute sinusitis is usually caused by a virus, lasts for about 2–3 weeks, and most people get better without antibiotics. Acute sinusitis usually follows a common cold, and symptoms for around 10 days or less are more likely to be associated with a cold rather than viral or bacterial acute sinusitis. Therefore, the committee agreed that an antibiotic prescription should not be offered to people presenting with acute sinusitis symptoms for around 10 days or less	FIN03: The majority of patients with sinusitis recover without antimicrobial therapy. Antimicrobial therapy should not be used to treat mild sinusitis because the disadvantages of treatment are more likely than its benefits
Urinary	Lower urinary tract infection	
	SCT03: Lower UTI is a self-limiting disease. If untreated, increased daytime urinary frequency lasts on average 6.3 days, dysuria 5.2 days, urgency 4.7 days, and patients report feeling generally unwell for on average 5.3 days, with moderately bad or worse symptoms for 3.8 days	NICE08: In most cases, managing lower UTIs will require antibiotic treatment. However, acute, uncomplicated lower UTI in non-pregnant women can be self-limiting and for some women delaying antibiotic treatment with a backup prescription to see if symptoms will resolve without antibiotic treatment may be an option
SSTI	Impetigo	
		COL01: Impetigo is in principle self-resolving, in a case where the infection is mild and there are no comorbidities in the patients, it may not require specific treatment

*Extended reporting: provided natural history information that included the duration of infection

**Basic reporting: provided minimal information, such as that the condition will typically spontaneously resolve

### Supporting references for natural history information reported in the guidelines

Of the 66 infections for which guidelines provided natural history information, supporting references were provided for most (91%, n = 60) (Table 2). Of the 125 references cited across the guidelines, 46.4% (n = 58) were from synthesised evidence (including 35 Cochrane systematic reviews, 16 systematic reviews of treatment effectiveness, 7 systematic reviews of cohort studies), 32.8% (n = 41) were primary studies (24 randomised controlled trials and 17 cohort studies) and 20.8% (n = 26) were references from other sources (such as other guidelines, editorials, position papers). There were variations in the cited references for the same conditions across different guidelines.

### Reporting of relevant antibiotic stewardship clinical strategies

#### Delayed antibiotic prescribing

A recommendation to use delayed antibiotic prescribing strategy was mentioned in 39% (n = 32) of the guidelines and for 34.2% (n = 39) of the eligible infections (see Additional file 1: G for details of delayed antibiotic prescribing recommendation by guidelines). It was most frequently recommended for respiratory tract infections (74.4%, n = 29), including in (92%, n = 11) of guidelines targeted at acute otitis media. For about half (51.3%, n = 20) of the infections, the recommendation for delayed prescribing included information about how long a patient should be advised to wait before deciding whether to fill the prescription. When timeframes were mentioned, there was variation across the guidelines. For example, for acute sinusitis the recommended waiting time ranged from 3 days (USA 04) to 7 days (NICE 07). Additional file 1: H contains verbatim examples of the reporting of delayed prescribing and the suggested waiting periods.

#### Shared decision making and patient decision aids

In 22% (n = 18) of the guidelines and 21.1% (n = 24) of the infections, it was recommended that shared decision making occur between clinicians and patients when deciding about antibiotic use (see Additional file 1: I for verbatim examples of recommendations). Using patient decision aids as a tool to support this conversation was mentioned in 18 of the 24 recommendations and an external link to an aid provided in all of these. Recommendations about shared decision making occurred most frequently for respiratory tract infections. Additional file 1: G provides details of shared decision making and decision aid recommendations by guidelines.

## Discussion

### Summary of the findings

In this systematic review of guidelines of acute infections commonly managed in primary care and with antibiotics, natural history information was reported for just over half of the eligible infections, most frequently for respiratory tract infections. For some infections (such as acute bronchitis, sinusitis, tonsilitis, conjunctivitis), natural history information was provided in all relevant guidelines, whereas for other infections (such as urinary tract infections and skin infections), it was rarely provided. In about two-thirds of the infections for which natural history was reported, quantitative information about the expected duration was provided. Delayed prescribing was recommended for about one-third of the assessed infections and shared decision making for about 21%.

### Strengths and limitations

To our knowledge, this is the first systematic review to investigate the reporting of natural history information in guidelines. The strength of this review lies in our comprehensive search strategy with no language restrictions. A limitation is that we may have missed guidelines that were not indexed in the databases searched or those that are not electronically accessible. Despite our supplementary search across well-known international guideline publishing organisations, we did not identify any eligible guidelines from Africa and only two from South America. Also, we did not assess the quality and appropriateness of the references used by the guidelines as support for the natural history information provided.

### Comparison with existing research

While guideline methodological quality has previously been assessed [35], there appears to be no other research that has explored the reporting of the natural history of acute infections. The methodological quality of guidelines in our sample is similar to findings of other reviews of acute infection guidelines [36, 37]. However, an assessment of methodological quality does not consider the thoroughness of the content included in the guidelines and natural history is a specific type of information that is not required for all conditions.

Some findings in our study can be compared with systematic reviews of guidelines that targeted specific acute infections. For example, a systematic review of European guidelines for the management of acute otitis media in children found 88% of the guidelines recommended a delayed antibiotic prescribing approach [36]. In our review, 92% of otitis media guidelines recommended delayed prescribing.

Despite international calls for antibiotic stewardship in primary care [9, 38, 39], across all the guidelines in our review, a minority recommended delayed prescribing and shared decision making. Our findings are similar to that of a systematic review of upper respiratory tract infections guidelines, where delayed prescribing for otitis media was recommended in only 3 of the 13 guidelines [37]. This is despite evidence of the usefulness of delayed prescribing in reducing antibiotic prescribing and use [40]. While awareness of shared decision making is increasing [41–43], it is a reasonably new strategy and may not yet be routinely incorporated into guidelines for acute infections. Furthermore, historically antibiotic stewardship strategies that have been promoted for primary care have included clinician education, diagnostic testing, audit and feedback, with less focus on delayed prescribing and shared decision making [44].

### Implications for practice

Many clinicians use guidelines from their country or professional organisation to stay up-to-date and the omission of natural history information in synthesised evidence resources such as guidelines is a missed opportunity to disseminate this information, which can inform clinician-patient conversations and decision-making. Natural history information is crucial for initiatives such as Choosing Wisely [45] campaigns in which patients and clinicians are encouraged to discuss “what happens if I don’t do anything?”. It is challenging to answer that question if research-informed natural history information is not readily available. Clinicians and patients can only know the difference a treatment, such as antibiotics, might make if they know what happens without that treatment. Knowing this may be an underutilised way of facilitating a conversation between clinicians and patients, managing patient expectations and misconceptions about the effectiveness of antibiotics, and reducing patient re-consultation for self-limiting acute infections [46, 47]. This may also contribute to reducing unnecessary prescribing and use of antibiotics in primary care.

While delayed prescribing and shared decision making are, for most of the conditions considered, generally appropriate, there are subgroups and circumstances when they may not be and the benefit-harm trade-off of antibiotics is altered. In such situations, the conversation should incorporate eliciting patients’ expectations and any misperceptions about the condition or treatment that they may have and providing patient education. Additionally, when using delayed antibiotic prescribing, clinicians need to explain the approach and the reason for it, carefully ensuring that a mixed message about antibiotic necessity is not conveyed.

### Implications for research

Across the guidelines, we found differences in the estimated duration of symptoms for many of the infections. This is most likely due to the differences in the design and quality of the studies chosen to provide the natural history information. For example, while the Australian Therapeutic Guidelines cite a Cochrane review [48] to support the natural history information about acute bronchitis, the Danish and Dutch guidelines cite different prospective cohort studies [49, 50] with different natural history duration for the same condition. The inclusion of natural history content is not a focus of guidelines and no guidance on how to search for, select, and report this information exists. Including natural history information in guidelines would be aided if more studies (primary and systematic reviews) with the primary aim of establishing the natural history of acute infections were conducted.

## Conclusion

Our review found an important gap in the inclusion and reporting of natural history information in guidelines, with just over half containing this information. Given the potential usefulness of natural history information in facilitating antibiotic stewardship strategies and the influence of guidelines on what treatment options clinicians present to patients, this is a missed opportunity to disseminate natural history information to clinicians and encourage its use in discussions with patients and informed decision-making.

## Supplementary Information

**Additional file 1. A1**. Search Strategy.** A2**. Additional guideline database search and number of guidelines identified and included from each.** B**. Inclusion and Exclusion criteria.** C**. List of included guidelines.** D**. List of excluded studies.** E**. Quality Assessment by AGREE II of 82 Evidence-based Guidelines.** F**. The reported duration of infections reported by only one guideline.** G**.Antibiotic Stewardship recommendations by guidelines for eligible infections*.** H**. Guidelines and examples of verbatim reporting of delayed prescribing.** I**. Examples of verbatim shared decision making recommendations in guidelines.

## Data Availability

All data generated and analysed during this study are included in this published article [and its additional information files].

## Abbreviations

PRISMA: Preferred Reporting Items for Systematic Reviews and Meta-analysis
TRIP: Turning Research into Practice
GIN: Guideline International Network
ARI: Acute respiratory infections
SSTI: Skin and soft tissue infection
UTI: Urinary tract infection
AGREE: Appraisal of Guidelines for Research and Evaluation

## References

[1] United Nations Interagency Coordination Group (IACG) on Antimicrobial Resistance 2019. Available from: https://www.who.int/publications/i/item/no-time-to-wait-securing-the-future-from-drug-resistant-infections.

[2] Llor C, Bjerrum L (2014). Antimicrobial resistance: risk associated with antibiotic overuse and initiatives to reduce the problem. Therapeutic Adv Drug Safety.

[3] World Health Organisation. Global action plan on antimicrobial resistance. 2015. Available from: https://www.who.int/publications/i/item/9789241509763.

[4] Bell BG, Schellevis F, Stobberingh E, Goossens H, Pringle M (2014). A systematic review and meta-analysis of the effects of antibiotic consumption on antibiotic resistance. BMC Infect Dis.

[5] Goossens H, Ferech M, Vander Stichele R, Elseviers M, Group EP (2005). Outpatient antibiotic use in Europe and association with resistance: a cross-national database study. Lancet (London, England)..

[6] McCullough AR, Pollack AJ, Hansen MP, Glasziou PP, Looke DF, Britt HC (2017). Antibiotics for acute respiratory infections in general practice: comparison of prescribing rates with guideline recommendations. Med J Aust.

[7] Sulis G, Adam P, Nafade V, Gore G, Daniels B, Daftary A (2020). Antibiotic prescription practices in primary care in low- and middle-income countries: a systematic review and meta-analysis. PLoS Med.

[8] Finley CR, Chan DS, Garrison S, Korownyk C, Kolber MR, Campbell S (2018). What are the most common conditions in primary care?. Can Fam Physician.

[9] Respiratory Tract Infections—Antibiotic Prescribing: Prescribing of Antibiotics for Self-Limiting Respiratory Tract Infections in Adults and Children in Primary Care London: National Institute for Health and Clinical Excellence (UK); 2008. Available from: http://www.ncbi.nlm.nih.gov/books/NBK53632/. 21698847

[10] Venekamp RP, Sanders SL, Glasziou PP, Del Mar CB, Rovers MM. Antibiotics for acute otitis media in children. Cochrane Database Syst Rev. 2015(6). 10.1002/14651858.CD000219.pub4 PMC7043305 26099233

[11] Principles of Epidemiology | Lesson 1—Section 9 2020. Available from: https://www.cdc.gov/csels/dsepd/ss1978/lesson1/section9.html.

[12] Graham ID, Harrison MB (2005). Evaluation and adaptation of clinical practice guidelines. Evid Based Nurs.

[13] Rosenfeld RM, Shiffman RN (2009). Clinical practice guideline development manual: a quality-driven approach for translating evidence into action. Otolaryngol Head Neck Surg.

[14] Del Mar C, Hoffmann T, Bakhit M (2022). How can general practitioners reduce antibiotic prescribing in collaboration with their patients?. Aust J Gen Pract.

[15] Hoffmann TC, Bakhit M, Del Mar C (2021). Uncomplicated urinary tract infection in women. BMJ (Online)..

[16] Bell KJL, McCullough A, Del Mar C, Glasziou P. What’s the uptake? Pragmatic RCTs may be used to estimate uptake, and thereby population impact of interventions, but better reporting of trial recruitment processes is needed. BMC Med Res Methodol. 2017;17(1):174–174. 10.1186/s12874-017-0443-0. 10.1186/s12874-017-0443-0 PMC5741884 29272994

[17] Stuart B, Hounkpatin H, Becque T, Yao G, Zhu S, Alonso-Coello P (2021). Delayed antibiotic prescribing for respiratory tract infections: individual patient data meta-analysis. BMJ.

[18] Hoffmann TC, Montori VM, Del Mar C (2014). The connection between evidence-based medicine and shared decision making. JAMA.

[19] Little P, Moore M, Kelly J, Williamson I, Leydon G, McDermott L (2014). Delayed antibiotic prescribing strategies for respiratory tract infections in primary care: pragmatic, factorial, randomised controlled trial. BMJ.

[20] Butler CC, Rollnick S, Kinnersley P, Tapper-Jones L, Houston H (2004). Communicating about expected course and re-consultation for respiratory tract infections in children: an exploratory study. Br J Gen Pract.

[21] Glasziou P (2002). The importance of prognostic research. Aust Fam Physician.

[22] Coxeter PD, Mar CD, Hoffmann TC (2017). Parents’ expectations and experiences of antibiotics for acute respiratory infections in primary care. Ann Fam Med.

[23] Hoffmann TC, Del Mar C (2015). Patients’ expectations of the benefits and harms of treatments, screening, and tests: a systematic review. JAMA Intern Med.

[24] Hoffmann TC, Del Mar C (2017). Clinicians’ expectations of the benefits and harms of treatments, screening, and tests: a systematic review. JAMA Intern Med.

[25] Gaarslev C, Yee M, Chan G, Fletcher-Lartey S, Khan R. A mixed methods study to understand patient expectations for antibiotics for an upper respiratory tract infection. Antimicrob Resist Infect Control. 2016;5. 10.1186/s13756-016-0134-3 PMC5072313 27777760

[26] Tonkin‐Crine SK, Tan PS, Hecke Ov, Wang K, Roberts NW, McCullough A, et al. Clinician‐targeted interventions to influence antibiotic prescribing behaviour for acute respiratory infections in primary care: an overview of systematic reviews. Cochrane Database Syst Rev. 2017(9). 10.1002/14651858.CD012252.pub2 PMC6483738 28881002

[27] Mitra A, Hannay D, Kapur A, Baxter G (2011). The natural history of acute upper respiratory tract infections in children. Prim Health Care Res Dev.

[28] Page MJ, McKenzie JE, Bossuyt PM, Boutron I, Hoffmann TC, Mulrow CD (2021). The PRISMA 2020 statement: an updated guideline for reporting systematic reviews. BMJ.

[29] Johnston A, Kelly SE, Hsieh S-C, Skidmore B, Wells GA (2019). Systematic reviews of clinical practice guidelines: a methodological guide. J Clin Epidemiol.

[30] Booth A, Clarke M, Dooley G, Ghersi D, Moher D, Petticrew M (2012). The nuts and bolts of PROSPERO: an international prospective register of systematic reviews. Syst Rev.

[31] Bigio J, MacLean E, Vasquez NA, Huria L, Kohli M, Gore G (2022). Most common reasons for primary care visits in low- and middle-income countries: a systematic review. PLOS Global Public Health.

[32] Cooke G, Valenti L, Glasziou P, Britt H (2013). Common general practice presentations and publication frequency. Aust Fam Physician.

[33] Brouwers MC, Kho ME, Browman GP, Burgers JS, Cluzeau F, Feder G (2010). AGREE II: advancing guideline development, reporting and evaluation in health care. CMAJ Can Med Assoc J journal de l’Association medicale canadienne..

[34] Bakhit M, Mar CD, Gibson E, Hoffmann T (2018). Shared decision making and antibiotic benefit-harm conversations: an observational study of consultations between general practitioners and patients with acute respiratory infections. BMC Fam Pract.

[35] Jiang M, Guan W jie, Fang Z fu, et al. A Critical Review of the Quality of Cough Clinical Practice Guidelines. Chest. 2016;150(4):777–88. 10.1016/j.chest.2016.04.028. 10.1016/j.chest.2016.04.028 27164291

[36] Suzuki HG, Dewez JE, Nijman RG, Yeung S (2020). Clinical practice guidelines for acute otitis media in children: a systematic review and appraisal of European national guidelines. BMJ Open.

[37] Zeng L, Zhang L, Hu Z, Ehle EA, Chen Y, Liu L (2014). Systematic review of evidence-based guidelines on medication therapy for upper respiratory tract infection in children with AGREE instrument. PLoS ONE.

[38] Centers for Disease Control and Prevention. Antibiotic Stewardship Statement | HICPAC | CDC 2019. Available from: https://www.cdc.gov/hicpac/recommendations/antibiotic-stewardship-statement.html.

[39] Spurling GKP, Del Mar CB, Dooley L, Clark J, Askew DA. Delayed antibiotic prescriptions for respiratory infections. Cochrane Database Syst Rev. 2017(9). 10.1002/14651858.CD004417.pub5 PMC6372405 28881007

[40] Spurling GKP, Del Mar CB, Dooley L, Clark J, Askew DA. Delayed antibiotic prescriptions for respiratory infections. Cochrane Database Syst Rev. 2017(9). 10.1002/14651858.CD004417.pub5 PMC6372405 28881007

[41] Bravo P, Härter M, McCaffery K, Giguère A, Hahlweg P, Elwyn G (2022). Editorial: 20 years after the start of international Shared Decision-Making activities: is it time to celebrate? Probably…. Z Evid Fortbild Qual Gesundhwes.

[42] Hoffmann TC, Légaré F, Simmons MB, McNamara K, McCaffery K, Trevena LJ (2014). Shared decision making: what do clinicians need to know and why should they bother?. Med J Aust.

[43] NICE guideline recommends shared decision making in all healthcare settings. PharmacoEconomics & outcomes news. 2021;(882):34–34.

[44] Hansen MP, Hoffmann TC, McCullough AR, van Driel ML, Del Mar CB (2015). Antibiotic resistance: what are the opportunities for primary care in alleviating the crisis?. Front Public Health.

[45] Levinson W, Born K, Wolfson D (2018). Choosing wisely campaigns: A work in progress. JAMA, J Am Med Assoc.

[46] Coxeter P, Del Mar CB, McGregor L, Beller EM, Hoffmann TC (2015). Interventions to facilitate shared decision making to address antibiotic use for acute respiratory infections in primary care. Cochrane Database Syst Rev.

[47] Little P, Stuart B, Hobbs FD, Butler CC, Hay AD, Delaney B (2014). Antibiotic prescription strategies for acute sore throat: a prospective observational cohort study. Lancet Infect Dis.

[48] Smith SM, Fahey T, Smucny J, Becker LA. Antibiotics for acute bronchitis. Cochrane Database Syst Rev. 2017(6). 10.1002/14651858.CD000245.pub4 PMC6481481 28626858

[49] Moore M, Little P, Rumsby K, Kelly J, Watson L, Warner G (2008). Predicting the duration of symptoms in lower respiratory tract infection. Br J Gen Pract.

[50] van Vugt SF, Butler CC, Hood K, Kelly MJ, Coenen S, Goossens H (2012). Predicting benign course and prolonged illness in lower respiratory tract infections: a 13 European country study. Fam Pract.

