# Correction to: Information about the natural history of acute infections commonly seen in primary care: a systematic review of clinical practice guidelines

**DOI:** 10.1186/s12879-022-07964-5

**Published:** 2023-01-09

**Authors:** Kwame Peprah Boaitey, Mina Bakhit, Natalia Krzyzaniak, Tammy C. Hofmann

**Affiliations:** Faculty of Health Sciences and Medicine, Institute for Evidence-Based Healthcare, Bond University, 14 University Dr, Robina, QLD 4229 Australia

**Correction to: BMC Infectious Diseases (2022) 22, 897.** https://doi.org/10.1186/s12879-022-07887-1

**Error 1:**

Following publication of the original article [1], the authors identified an error in Box 1. The correct box is given below.
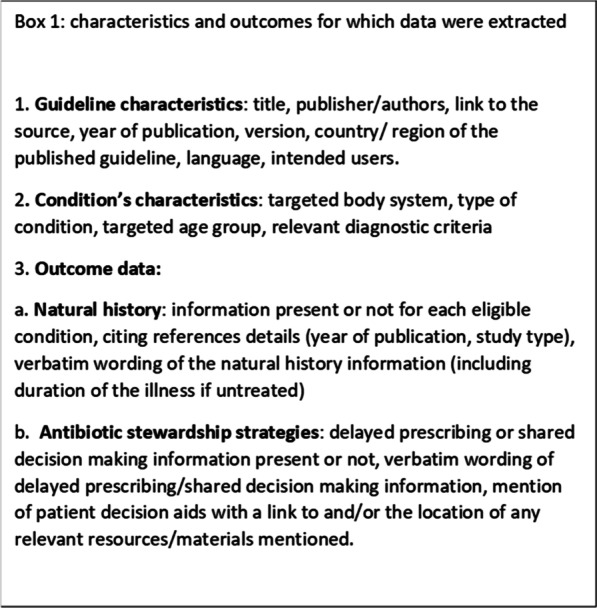


**Error 2:**

Following publication of the original article [1], the authors identified an error in Fig. 2. The correct figure is given below.

**Fig. 2:**
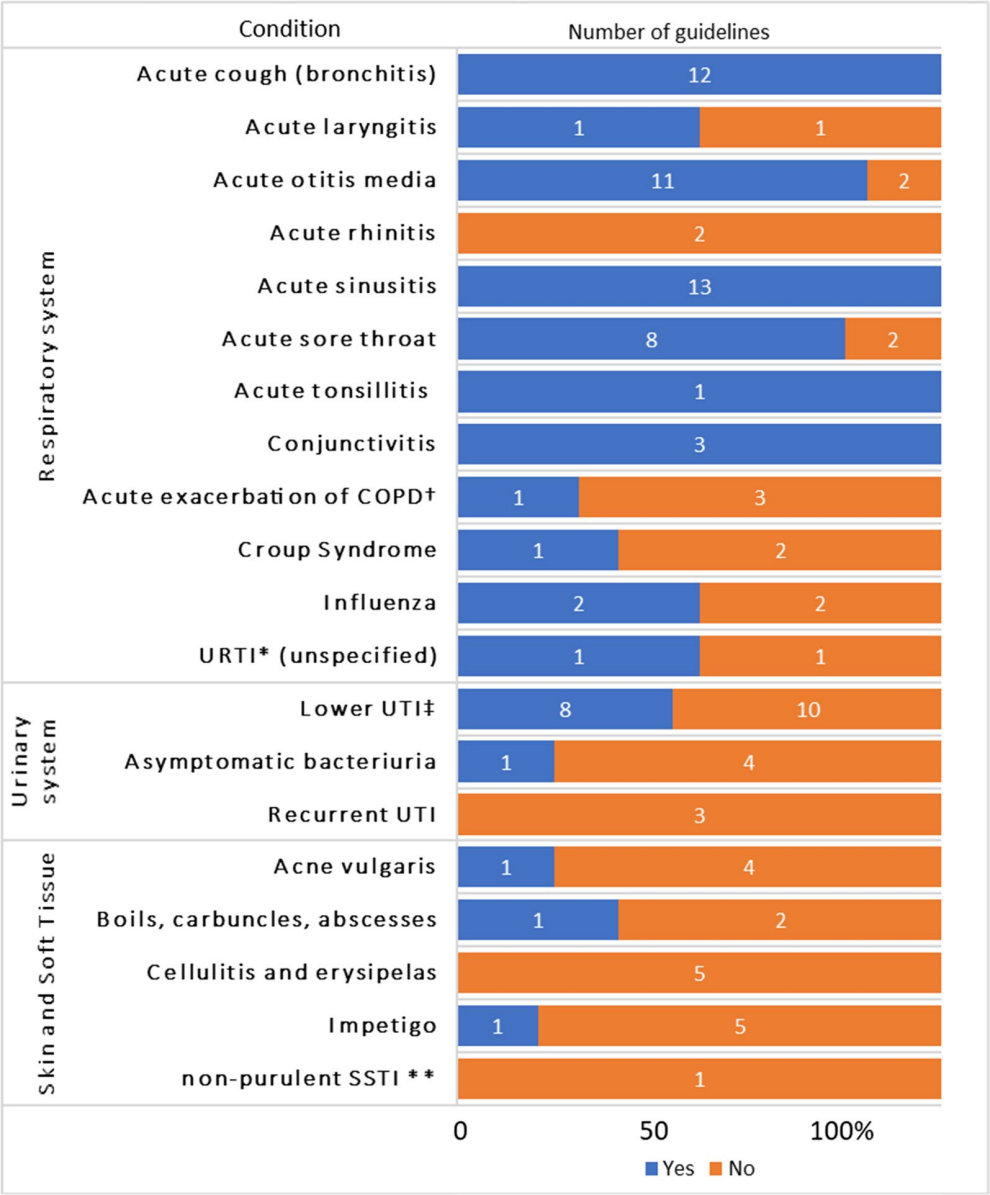
Percentage of guidelines that, for each of the acute infections, reported the natural history information (number of guidelines addressing each condition shown within each bar). ^†^Chronic Obstructive Pulmonary Disease, *Upper respiratory tract infection, ^‡^Urinary tract infection, **Skin and soft tissue infection
