# Supplementary material for: Information about the natural history of acute infections commonly seen in primary care: a systematic review of clinical practice guidelines
Source: BMC Infect Dis. 2022 Dec 1;22:897. doi: 10.1186/s12879-022-07887-1 (PMC9714117; doi:10.1186/s12879-022-07887-1)
Supplement: Supplementary file 1 — Additional file 1. A1. Search Strategy. A2. Additional guideline database search and number of guidelines identified and included from each. B. Inclusion and Exclusion criteria. C. List of included guidelines. D. List of excluded studies. E. Quality Assessment by AGREE II of 82 Evidence-based Guidelines. F. The reported duration of infections reported by only one guideline. G.Antibiotic Stewardship recommendations by guidelines for eligible infections*. H. Guidelines and examples of verbatim reporting of delayed prescribing. I. Examples of verbatim shared decision making recommendations in guidelines. [file 12879_2022_7887_MOESM1_ESM.docx]

**Supplementary materials**

**Additional file A1: Search Strategy**

1. PubMed, Embase and CINAHL) run 22/02/2021.

Results: With Dups = 3320: No dups = 2786

**SSTI:** ("Practice Guideline"[Publication Type] OR “Clinical guideline”[ti] OR “Clinical guidelines”[ti] OR “Practice guideline”[ti] OR “Practice guidelines”[ti] OR “Clinical practice guideline”[ti] OR “Clinical practice guidelines”[ti])AND(Erysipelas[Mesh] OR Erysipelas[tiab] OR "Skin Diseases, Infectious"[Mesh] OR Cellulitis[Mesh] OR Cellulitis[tiab] OR Impetigo[tiab] OR Impetigo[Mesh] OR "Soft Tissue Infections"[Mesh] OR "Holy fire”[tiab] OR "st anthony's fire"[tiab] OR "Soft Tissue Infections"[tiab] OR "Soft Tissue Infection"[tiab] OR "Skin infections"[tiab] OR "Skin infection"[tiab] OR ((Staphylococcus[Mesh] OR staphylococcus[tiab] OR Streptococcus[Mesh] OR streptococcus[tiab]) AND (Skin[Mesh] OR Skin[tiab])))

**UTI:** ("Practice Guideline"[Publication Type] OR “Clinical guideline”[ti] OR “Clinical guidelines”[ti] OR “Practice guideline”[ti] OR “Practice guidelines”[ti] OR “Clinical practice guideline”[ti] OR “Clinical practice guidelines”[ti])AND ("Urinary Tract Infections"[Mesh] OR “Urinary Tract Infections”[tiab] OR “Urinary Tract Infection”[tiab] OR UTIs[tiab] OR UTI[tiab] OR Cystitis[tiab])

**ARI:**("Practice Guideline"[Publication Type] OR “Clinical guideline”[ti] OR “Clinical guidelines”[ti] OR “Practice guideline”[ti] OR “Practice guidelines”[ti] OR “Clinical practice guideline”[ti] OR “Clinical practice guidelines”[ti])AND("Respiratory Tract Infections"[Mesh] OR Nasopharyngitis[Mesh] OR Rhinitis[Mesh] OR Sinusitis[Mesh] OR Pharyngitis[Mesh] OR Laryngitis[Mesh] OR bronchitis[Mesh] OR bronchiolitis[Mesh] OR "bronchiolitis, viral"[Mesh] OR Pleurisy[Mesh] OR Cough[Mesh] OR Sneezing[Mesh] OR Pneumonia[Mesh]  OR "Respiratory Sounds"[Mesh] OR  "Otitis Media"[Mesh] OR  Earache[Mesh] OR "Influenza, Human"[Mesh] OR "Common Cold"[Mesh]  OR “Respiratory infection”[tiab] OR “Respiratory infections”[tiab] OR “Respiratory Tract Infections"[tiab] OR “Respiratory Tract Infection"[tiab] OR “Respiratory inflammation”[tiab] OR “Respiratory tract inflammation”[tiab] OR urti[tiab] OR lrti[tiab] OR ari[tiab] OR  nasopharyngitis[tiab] OR rhinopharyngitis[tiab] OR sinusitis[tiab] OR nasosinusitis[tiab] OR rhinosinusitis[tiab] OR rhinitis[tiab] OR rhinorrhoea[tiab]  OR rhinorrhea[tiab] OR pharyngitis[tiab] OR "sore throat"[tiab] OR Tonsillitis[tiab] OR laryngitis[tiab] OR croup[tiab] OR pseudocroup[tiab]  OR tracheobronchitis[tiab] OR laryngotracheobronchitis[tiab] OR bronchitis[tiab] OR bronchiolitis[tiab] OR pneumonia[tiab] OR pleuropneumonia[tiab] OR bronchopneumonia[tiab] OR pleurisy[tiab] OR cough[tiab] OR Wheeze[tiab] OR Wheezing[tiab] OR "otitis media"[tiab] OR aom[tiab] OR ome[tiab] OR earache[tiab] OR influenza[tiab] OR flu[tiab] OR "common cold"[tiab] OR "common colds"[tiab])

TRIP search: Results: With Dups = 5812: No dups = 4781 Run 08/05/21

<https://www.tripdatabase.com/>

("Respiratory Tract Infections" OR Nasopharyngitis OR Rhinitis OR Sinusitis OR Pharyngitis OR Laryngitis OR bronchitis OR bronchiolitis OR Pleurisy OR Cough OR Sneezing OR Pneumonia OR "Respiratory Sounds" OR "Otitis Media" OR Earache OR "Influenza, Human" OR "Common Cold" OR Conjunctivitis OR "Respiratory infection" OR "Respiratory infections" OR "Respiratory Tract Infections" OR "Respiratory Tract Infection" OR "Respiratory inflammation" OR "Respiratory tract inflammation" OR urti OR lrti OR ari OR nasopharyngitis OR rhinopharyngitis OR sinusitis OR nasosinusitis OR rhinosinusitis OR rhinitis OR rhinorrhoea OR rhinorrhea OR pharyngitis OR "sore throat" OR Tonsillitis OR laryngitis OR croup OR pseudocroup OR tracheobronchitis OR laryngotracheobronchitis OR bronchitis OR bronchiolitis OR pneumonia OR pleuropneumonia OR bronchopneumonia OR pleurisy OR cough OR Wheeze OR Wheezing OR "otitis media" OR aom OR ome OR earache OR influenza OR flu OR "common cold" OR "common colds" OR Conjunctivitis)

(Erysipelas OR Erysipelas OR Cellulitis OR Cellulitis OR Impetigo OR Impetigo OR "Soft Tissue Infections" OR "Soft Tissue Infections" OR "Soft Tissue Infection" OR "Skin infections" OR "Skin infection")

**Additional file A2:** **Additional guideline database search and number of guidelines identified and included from each**

| **Country** | **Developer** | **Website** | **Guidelines included** |
| --- | --- | --- | --- |
| Netherland | Dutch College of General Practitioners (NHG) | https://richtlijnen.nhg.org/ | 7 |
| United Kingdom | National Institute for Health and Care Excellence (NICE) | https://www.nice.org.uk/ | 1 |
| Scotland | The Scottish Intercollegiate Guidelines Network (SIGN) | https://www.sign.ac.uk/ | 1 |
| Denmark | Danish Society for General Medicine (DSAM) | https://www.dsam.dk/publikationer/ | 1 |
| Australia | Therapeutic guideline Australia* | https://tgldcdp.tg.org.au/etgcomplete | 1 |
| Australia | National Health and Medicine Research Council (NHMRC) | https://www.nhmrc.gov.au/research-policy/guideline-development | 0 |
| Finland | Finnish Medical Society Duodecim | https://www.kaypahoito.fi/en/summaries | 5 |
| Germany | Association of Scientific Medical Societies (AWMF) | https://www.awmf.org/leitlinien/leitlinien-suche.html | 2 |
| Sweden | Swedish National Board for Medical and Social Evaluation | https://www.sbu.se/ | 0 |
| Canada | CPG Infobase: Clinical Practice Guidelines | https://joulecma.ca/cpg/homepage | 0 |
| Italy | National Guideline System | https://www.iss.it/web/iss-en/national-guidelines-system-snlg- | 0 |
| Switzerland | Swiss Medical Association \| FMH | https://www.fmh.ch/ | 0 |
| France | National agency for accreditation and assessment of health | http://www.anaes.fr/ | 0 |
| Spain | Ministry of health | https://www.sanidad.gob.es/ | 0 |
| Spain | Guia Salud | https://portal.guiasalud.es/gpc/?_sfm_wpcf-estado=1 | 0 |
| New Zealand | Ministry of health | https://www.health.govt.nz/about-ministry/ministry-health-websites/new-zealand-guidelines-group | 0 |
| Norway | The Norwegian Board of Health Supervision | https://www.helsetilsynet.no/ | 0 |
| WHO | World Health Organisation | https://www.who.int/publications/who-guidelines | 1 |
| Belgium | Ebpracticenet (BE) Working Group Development of Primary Care Guidelines | Guideline International network | 2 |
| Qatar | Ministry of health | Guideline International network | 2 |
| United States of America | Centers for Disease Prevention and Control | https://www.cdc.gov/antibiotic-use/ear-infection.html | 0 |
| China | China National Knowledge Infrastructure | https://en.cnki.com.cn// | 0 |
|  |  |  |  |

*An online-based guideline with paid subscription

**Additional file B : Inclusion and Exclusion criteria**

| **Inclusion criteria** |
| --- |
| 1. Acute respiratory infections including acute otitis media, sore throat, pharyngitis, tonsillitis, common cold, sinusitis, laryngitis, acute cough, acute bronchitis, bronchiolitis middle ear inflammatory conditions |
| 1. Rhinosinusitis, rhinitis, group A streptococcal infections, streptococcal pharyngitis |
| 1. Influenza-like illnesses including seasonal influenza |
| 1. Lower urinary tract infections, cystitis, acute bladder infection, recurrent UTI |
| 1. Skin and soft tissue infections, cellulitis, acne, impetigo, boils, carbuncles, ecthyma |
| 1. All forms of conjunctival conditions |
| 1. Management of croup |
| 1. Urinary tract infections in non-pregnant women |
| 1. Acute exacerbation of COPD |
| **Exclusion criteria** |

| 1. Covid-19 pandemic, avian flu, severe acute respiratory syndrome |
| --- |
| 1. Flu and influenza vaccination and immunization recommendations |
| 1. Hospital-acquired infections |
| 1. Chronic respiratory infections (e.g., chronic cough, COPD, chronic otitis media, chronic sinusitis, otitis media with effusion) |
| 1. Severe infections (e.g., meningitis, necrotizing tissue infections) |
| 1. Lower respiratory tract infections (e.g., pneumonia) |
| 1. Psoriasis, Leishmaniasis, Genital warts, herpes |
| 1. Guidelines targeting specific high-risk groups (e.g., immunocompromised patients, pregnant women, neurological disorders) |
| 1. Guidelines on diagnostic evaluation without a recommendation for treatment and management |
| 1. Guidelines targeting the use of specific therapies (e.g., immunotherapy, corticosteroids, herbal remedies) |

**Additional file C: List of included guidelines**

|  | Author | Date | Guideline Title | Journal | Code |
| --- | --- | --- | --- | --- | --- |
|  | Alberta Health Services | 2015 | Urinary Tract Infection - Long Term Care Facilities | Accelerating Change Transformation Team | CAN05 |
|  | American Academy of Dermatology | 2016 | Acne clinical guideline | American Academy of Dermatology | USA12 |
|  | American Academy of Family Physicians | 2013 | Diagnosis and Management of Acute Otitis Media | American Academy of Family Physicians | USA17 |
|  | American Academy of Pediatrics | 2013 | Clinical Practice Guideline for the Diagnosis and Management of Acute Bacterial Sinusitis in Children Aged 1 to 18 Years | American Academy of Pediatrics | USA15 |
|  | American Thoracic Society | 2017 | Management of COPD Exacerbations: An Official ERS/ATS Clinical Practice Guideline | American Thoracic Society | INT05 |
|  | Anger, J., Lee, U., Ackerman, A. L., Chou, R., Chughtai, B., Clemens, J. Q., Hickling, D., Kapoor, A., Kenton, K. S., Kaufman, M. R., Rondanina, M. A., Stapleton, A., Stothers, L. and Chai, T. C. | 2019 | Recurrent Uncomplicated Urinary Tract Infections in Women: AUA/CUA/SUFU Guideline | The Journal of Urology | USA20 |
|  | Australia Therapeutic Guidelines | 2019 | TG Complete | Therapeutic Guidelines | AUS03 |
|  | Australian Government | 2014 | Acute Otitis Media in Children | SA Health Paediatric Clinical Practice Guidelines | AUS01 |
|  | Australian Government | 2018 | Croup | SA Health Paediatric Clinical Practice Guidelines | AUS02 |
|  | Canadian Society of Obstetricians and Gynaecologists | 2017 | Recurrent Urinary Tract Infection | Journal of Society of Obstetricians and Gynaecologists of Canada | CAN04 |
|  | Caron, F., Galperine, T., Flateau, C., Azria, R., Bonacorsi, S., Bruyère, F., Cariou, G., Clouqueur, E., Cohen, R., Doco-Lecompte, T., Elefant, E., Faure, K., Gauzit, R., Gavazzi, G., Lemaitre, L., Raymond, J., Senneville, E., Sotto, A., Subtil, D., Trivalle, C., Merens, A. and Etienne, M. | 2018 | Practice guidelines for the management of adult community-acquired urinary tract infections | Médecine et Maladies Infectieuses | FRA01 |
|  | Choi, W. S., Lee, J., Lee, H. Y., Baek, J. H., Kim, Y. K., Kee, S. Y., Jeong, H. W., Kim, Y. K., Song, J. Y., Wie, S. H. and Lee, J. S. | 2012 | Clinical practice guideline for antiviral treatment and chemoprophylaxis of seasonal influenza | Infection and Chemotherapy | KOR04 |
|  | Chow, A. W., Benninger, M. S., Brook, I., Brozek, J. L., Goldstein, E. J., Hicks, L. A., Pankey, G. A., Seleznick, M., Volturo, G., Wald, E. R. and File, T. M., Jr. | 2012 | IDSA clinical practice guideline for acute bacterial rhinosinusitis in children and adults | Clinical Infectious Diseases | USA04 |
|  | Danish Society of General Medicine | 2014 | Respiratory tract infections | Danish Society of General medicine | DEN02 |
|  | de Cueto, M., Aliaga, L., Alós, J. I., Canut, A., Los-Arcos, I., Martínez, J. A., Mensa, J., Pintado, V., Rodriguez-Pardo, D., Yuste, J. R. and Pigrau, C. | 2017 | Executive summary of the diagnosis and treatment of urinary tract infection: Guidelines of the Spanish Society of Clinical Microbiology and Infectious Diseases (SEIMC) | Enferm Infecc Microbiol Clin | SPN01 |
|  | Desrosiers, M., Evans, G.A., Keith, P.K. et al. | 2011 | Canadian clinical practice guidelines for acute and chronic rhinosinusitis | CPG Infobase | CAN02 |
|  | Dutch College of General Practitioners | 2014 | Acute otitis media in children | Dutch College of General Practitioners (NHG) | NED05 |
|  | Dutch College of General Practitioners | 2014 | Acute Rhinosinusitis | Dutch College of General Practitioners (NHG) | NED06 |
|  | Dutch College of General Practitioners | 2015 | Acute sore throat | Dutch College of General Practitioners (NHG) | NED03 |
|  | Dutch College of General Practitioners | 2017 | Red eye and Trauma | Dutch College of General Practitioners (NHG). | NED04 |
|  | Dutch College of General Practitioners | 2017 | Acne | Dutch College of General Practitioners (NHG) | NED07 |
|  | Dutch College of General Practitioners | 2020 | Urinary Tract Infections | Dutch College of General Practitioners (NHG) | NED08 |
|  | Dutch College of General Practitioners | 2013 | Acute cough | Dutch College of General Practitioners (NHG) | NED01 |
|  | European Dermatology Forum | 2016 | Guideline for the Treatment of Acne | Accelerating Change Transformation Team | INT04 |
|  | Ebpracticenet (BE) Working Group | 2016 | Guideline Cystitis in women | Ebpracticenet (BE) Working Group Development of Primary Care Guidelines | BEL02 |
|  | Ebpracticenet (BE) Working Group | 2017 | Sore throat | Ebpracticenet (BE) Working Group Development of Primary Care Guidelines | BEL01 |
|  | Finnish Medical Society Duodecim | 2015 | Lower respiratory tract infections (adults) | Finnish Medical Society Duodecim | FIN05 |
|  | Finnish Medical Society Duodecim | 2017 | Acute otitis media | Finnish Medical Society Duodecim | FIN02 |
|  | Finnish Medical Society Duodecim | 2018 | Acute sinusitis | Finnish Medical Society Duodecim | FIN03 |
|  | Finnish Medical Society Duodecim | 2020 | Pharyngitis | Finnish Medical Society Duodecim | FIN04 |
|  | Finnish Medical Society Duodecim | 2021 | Urinary tract infections | Finnish Medical Society Duodecim | FIN06 |
|  | German Association of Scientific Medical Societies | 2014 | Earache | Association of Scientific Medical Societies, Germany | GER07 |
|  | German Society for Family and General Medicine | 2020 | Sore throat | German Society for family medicine and General medicine | GER08 |
|  | Gupta, K., Hooton, T. M., Naber, K. G., Wullt, B., Colgan, R., Miller, L. G., Moran, G. J., Nicolle, L. E., Raz, R., Schaeffer, A. J. and Soper, D. E. | 2011 | International clinical practice guidelines for the treatment of acute uncomplicated cystitis and pyelonephritis in women: A 2010 update by the Infectious Diseases Society of America and the European Society for Microbiology and Infectious Diseases | Clin Infect Dis | USA03 |
|  | Haute Autorite de sante. Electronic address, contact sbpp has-sante fr | 2019 | Management of common bacterial skin infections | J Med Vasc | FRA02 |
|  | Hayashi, T., Kitamura, K., Hashimoto, S., Hotomi, M., Kojima, H., Kudo, F., Maruyama, Y., Sawada, S., Taiji, H., Takahashi, G., Takahashi, H., Uno, Y. and Yano, H. | 2020 | Clinical practice guidelines for the diagnosis and management of acute otitis media in children—2018 update | Auris Nasus Larynx | JPN03 |
|  | French National Authority for Health, | 2016 | Paediatric sinusitis | HAS Guidelines | FRA03 |
|  | Heidemann, C. H., Lous, J., Berg, J., Christensen, J. J., Håkonsen, S. J., Jakobsen, M., Johansen, C. J., Nielsen, L. H., Hansen, M. P., Poulsen, A., Schousboe, L. P., Skrubbeltrang, C., Vind, A. B. and Homøe, P. | 2016 | Danish guidelines on management of otitis media in preschool children | Int J Pediatr Otorhinolaryngol | DEN01 |
|  | Holzinger, F. and Beck, S. | 2014 | Diagnostics and treatment of cough | Dtsch Med Wochenschr | GER02 |
|  | Institute for Clinical Systems Improvement | 2017 | Respiratory Illness in Children and Adults, Diagnosis and Treatment of | Institute for Clinical Systems Improvement | USA21 |
|  | Kang, C. I., Kim, J., Park, D. W., Kim, B. N., Ha, U. S., Lee, S. J., Yeo, J. K., Min, S. K., Lee, H. and Wie, S. H. | 2018 | Clinical Practice Guidelines for the Antibiotic Treatment of Community-Acquired Urinary Tract Infections | Infect Chemother | KOR02 |
|  | Korean Society of infectious diseases | 2011 | Clinical guideline for the diagnosis and treatment of urinary tract infections: Asymptomatic bacteriuria, uncomplicated & complicated urinary tract infections, bacterial prostatitis | Infection and Chemotherapy | KOR06 |
|  | Kranz, J., Schmidt, S., Lebert, C., Schneidewind, L., Mandraka, F., Kunze, M., Helbig, S., Vahlensieck, W., Naber, K., Schmiemann, G. and Wagenlehner, F. M. | 2018 | The 2017 Update of the German Clinical Guideline on Epidemiology, Diagnostics, Therapy, Prevention, and Management of Uncomplicated Urinary Tract Infections in Adult Patients: Part 1 | Urol Int | GER04 |
|  | Kwak, Y. G., Choi, S. H., Kim, T., Park, S. Y., Seo, S. H., Kim, M. B. and Choi, S. H. | 2017 | Clinical Guidelines for the Antibiotic Treatment for Community-Acquired Skin and Soft Tissue Infection | Infect Chemother | KOR05 |
|  | Lai, K., Shen, H., Zhou, X., Qiu, Z., Cai, S., Huang, K., Wang, Q., Wang, C., Lin, J., Hao, C., Kong, L., Zhang, S., Chen, Y., Luo, W., Jiang, M., Xie, J. and Zhong, N. | 2018 | Clinical Practice Guidelines for Diagnosis and Management of Cough-Chinese Thoracic Society (CTS) Asthma Consortium | J Thorac Dis | CHI01 |
|  | Lee, H. J., Park, S. K., Choi, K. Y., Park, S. E., Chun, Y. M., Kim, K. S., Park, S. N., Cho, Y. S., Kim, Y. J. and Kim, H. J. | 2012 | Korean clinical practice guidelines: Otitis media in children | Journal of Korean Medical Science | KOR01 |
|  | Mikasa, K., Aoki, N., Aoki, Y., Abe, S., Iwata, S., Ouchi, K., Kasahara, K., Kadota, J., Kishida, N., Kobayashi, O., Sakata, H., Seki, M., Tsukada, H., Tokue, Y., Nakamura-Uchiyama, F., Higa, F., Maeda, K., Yanagihara, K. and Yoshida, K. | 2016 | JAID/JSC Guidelines for the Treatment of Respiratory Infectious Diseases: The Japanese Association for Infectious Diseases/Japanese Society of Chemotherapy - The JAID/JSC Guide to Clinical Management of Infectious Disease/Guideline-preparing Committee Respiratory Infectious Disease WG | J Infect Chemother | JPN02 |
|  | Ministry of Health, Malaysia | 2019 | Management of Rhinosinusitis in Adolescents and Adults | Ministry of Health, Malaysia | MAL01 |
|  | National Institute for Health and Clinical Excellence | 2017 | Sinusitis (acute): antimicrobial prescribing | National Institute for Health and Clinical Excellence - Clinical Guidelines | NICE07 |
|  | National Institute for Health and Clinical Excellence | 2018 | Chronic obstructive pulmonary disease (acute exacerbation): antimicrobial prescribing | National Institute for Health and Clinical Excellence - Clinical Guidelines | NICE03 |
|  | National Institute for Health and Clinical Excellence | 2018 | Otitis media (acute): antimicrobial prescribing | National Institute for Health and Clinical Excellence - Clinical Guidelines | NICE06 |
|  | National Institute for Health and Clinical Excellence | 2018 | Sore throat (acute): antimicrobial prescribing | National Institute for Health and Clinical Excellence - Clinical Guidelines | NICE10 |
|  | National Institute for Health and Clinical Excellence | 2018 | Urinary tract infection (lower): antimicrobial prescribing | National Institute for Health and Clinical Excellence - Clinical Guidelines | NICE08 |
|  | National Institute for Health and Clinical Excellence | 2018 | Urinary tract infection (recurrent): antimicrobial prescribing | National Institute for Health and Clinical Excellence - Clinical Guidelines | NICE09 |
|  | National Institute for Health and Clinical Excellence | 2019 | Cellulitis and erysipelas: antimicrobial prescribing | National Institute for Health and Clinical Excellence - Clinical Guidelines | NICE02 |
|  | National Institute for Health and Clinical Excellence | 2019 | Cough (acute): antimicrobial prescribing | National Institute for Health and Clinical Excellence - Clinical Guidelines | NICE04 |
|  | National Institute for Health and Clinical Excellence | 2020 | Impetigo: antimicrobial prescribing | National Institute for Health and Clinical Excellence - Clinical Guidelines | NICE05 |
|  | National Institute for Health and Clinical Excellence | 2021 | Acne vulgaris: management | National Institute for Health and Clinical Excellence - Clinical Guidelines | NICE11 |
|  | Nicolle, L. E., Gupta, K., Bradley, S. F., Colgan, R., DeMuri, G. P., Drekonja, D., Eckert, L. O., Geerlings, S. E., Köves, B., Hooton, T. M., Juthani-Mehta, M., Knight, S. L., Saint, S., Schaeffer, A. J., Trautner, B., Wullt, B. and Siemieniuk, R. | 2019 | Clinical Practice Guideline for the Management of Asymptomatic Bacteriuria: 2019 Update by the Infectious Diseases Society of America | Clin Infect Dis | USA08 |
|  | Qatar Ministry of health | 2019 | National Clinical Guidelines; The Diagnosis and Management of the Common Cold in Adults and Children. | Ministry of health, Qatar | QAT01 |
|  | Qatar Ministry of Health | 2020 | National Clinical Guidelines; The Diagnosis and Management of Lower Urinary Tract Infection in Adult Women | Ministry of health, Qatar | QAT02 |
|  | Pelucchi, C., Grigoryan, L., Galeone, C., Esposito, S., Huovinen, P., Little, P. and Verheij, T. | 2012 | Guideline for the management of acute sore throat | Clin Microbiol Infect | INT02 |
|  | Perepanova, T. S. | 2016 | The 2015 Federal Clinical Guidelines for Antimicrobial Therapy and Prevention of Infections of the Kidney, Urinary Tract, and Male Genitals | Ter Arkh | RUS01 |
|  | Peters AT, Spector S, Hsu J, Hamilos DL, Baroody FM, Chandra RK, Grammer LC, Kennedy DW, Cohen NA, Kaliner MA, Wald ER, Karagianis A, Slavin RG | 2014 | Diagnosis and management of rhinosinusitis: a practice parameter update | Pediatrics | USA05 |
|  | Rosenfeld, R. M., Piccirillo, J. F., Chandrasekhar, S. S., Brook, I., Ashok Kumar, K., Kramper, M., Orlandi, R. R., Palmer, J. N., Patel, Z. M., Peters, A., Walsh, S. A. and Corrigan, M. D. | 2015 | Clinical practice guideline (update): adult sinusitis | Otolaryngol Head Neck Surg | USA10 |
|  | Scadding, G. K., Kariyawasam, H. H., Scadding, G., Mirakian, R., Buckley, R. J., Dixon, T., Durham, S. R., Farooque, S., Jones, N., Leech, S., Nasser, S. M., Powell, R., Roberts, G., Rotiroti, G., Simpson, A., Smith, H. and Clark, A. T. | 2017 | BSACI guideline for the diagnosis and management of allergic and non-allergic rhinitis (Revised Edition 2017 | First edition 2007) | INT01 |
|  | Schöfer, H., Bruns, R., Effendy, I., Hartmann, M., Jappe, U., Plettenberg, A., Reimann, H., Seifert, H., Shah, P., Sunderkötter, C., Weberschock, T., Wichelhaus, T. A. and Nast, A. | 2011 | Diagnosis and treatment of Staphylococcus aureus infections of the skin and mucous membranes | J Dtsch Dermatol Ges | GER03 |
|  | Scottish Intercollegiate Network | 2020 | Management of suspected bacterial lower urinary tract infection in adult women | SIGN | SCT03 |
|  | Shulman, S. T., Bisno, A. L., Clegg, H. W., Gerber, M. A., Kaplan, E. L., Lee, G., Martin, J. M. and Van Beneden, C. | 2012 | Clinical practice guideline for the diagnosis and management of group A streptococcal pharyngitis: 2012 update by the Infectious Diseases Society of America | Clin Infect Dis | USA07 |
|  | Smith, M. P., Lown, M., Singh, S., Ireland, B., Hill, A. T., Linder, J. A. and Irwin, R. S. | 2020 | Acute Cough Due to Acute Bronchitis in Immunocompetent Adult Outpatients: CHEST Expert Panel Report | Chest | USA11 |
|  | Stevens, D. L., Bisno, A. L., Chambers, H. F., Dellinger, E. P., Goldstein, E. J. C., Gorbach, S. L., Hirschmann, J. V., Kaplan, S. L., Montoya, J. G. and Wade, J. C. | 2014 | Practice guidelines for the diagnosis and management of skin and soft tissue infections: 2014 update by the infectious disease’s society of America | Clinical Infectious Diseases | USA02 |
|  | Stuck, B. A., Beule, A., Jobst, D., Klimek, L., Laudien, M., Lell, M., Vogl, T. J. and Popert, U. | 2018 | Guideline for "rhinosinusitis"-long version: S2k guideline of the German College of General Practitioners and Family Physicians and the German Society for Oto-Rhino-Laryngology, Head and Neck Surgery | Hno | GER01 |
|  | Tapiainen, T., Aittoniemi, J., Immonen, J., Jylkkä, H., Meinander, T., Nuolivirta, K., Peltola, V., Salo, E., Seuri, R., Walle, S. M. and Korppi, M. | 2016 | Finnish guidelines for the treatment of laryngitis, wheezing bronchitis and bronchiolitis in children | Acta Paediatr | FIN01 |
|  | Uyeki, T. M., Bernstein, H. H., Bradley, J. S., Englund, J. A., File, T. M., Fry, A. M., Gravenstein, S., Hayden, F. G., Harper, S. A., Hirshon, J. M., Ison, M. G., Johnston, B. L., Knight, S. L., McGeer, A., Riley, L. E., Wolfe, C. R., Alexander, P. E. and Pavia, A. T. | 2019 | Clinical Practice Guidelines by the Infectious Diseases Society of America: 2018 Update on Diagnosis, Treatment, Chemoprophylaxis, and Institutional Outbreak Management of Seasonal Influenza | Clinical Infectious Diseases | USA06 |
|  | Valderrama-Beltrán, S., Cortés, J. A., Caro, M. A., Cely-Andrade, L., Osorio-Pinzón, J. V., Gualtero, S. M., Berrio-Medina, I., Rodriguez, J. Y., Granada-Copete, A. M., Guevara, F., Sefair, C., Leal, A. L., Jiménez, J. N. and Álvarez-Moreno, C. | 2019 | Clinical practice guidelines for the diagnosis and management of skin and soft tissue infections in Colombia | Infectio | COL01 |
|  | Varu, D. M., Rhee, M. K., Akpek, E. K., Amescua, G., Farid, M., Garcia-Ferrer, F. J., Lin, A., Musch, D. C., Mah, F. S. and Dunn, S. P. | 2019 | Conjunctivitis Preferred Practice Pattern® | Ophthalmology | USA01 |
|  | Villarraga, J. D. A., Parra, J. D. I., Diaz, D. A., Cardenas, A. M., Chavarriaga, J. and Godoy, M. P. | 2018 | Clinical practice guideline for urinary tract infection in adults | Urologia Colombiana | COL02 |
|  | Windfuhr, J. P., Toepfner, N., Steffen, G., Waldfahrer, F. and Berner, R. | 2016 | Clinical practice guideline: tonsillitis I. Diagnostics and nonsurgical management | Eur Arch Otorhinolaryngol | GER05 |
|  | Woodhead, M., Blasi, F., Ewig, S., Garau, J., Huchon, G., Ieven, M., Ortqvist, A., Schaberg, T., Torres, A., van der Heijden, G., Read, R. and Verheij, T. J. | 2011 | Guidelines for the management of adult lower respiratory tract infections--full version | Clin Microbiol Infect | INT03 |
|  | World Health Organisation | 2012 | Recommendations for management of common childhood conditions, | World Health Organisation | WHO01 |
|  | Yamamoto, S., Ishikawa, K., Hayami, H., Nakamura, T., Miyairi, I., Hoshino, T., Hasui, M., Tanaka, K., Kiyota, H. and Arakawa, S. | 2016 | The JAID/JSC Guidelines to Clinical Management of Infectious Diseases (Urinary tract infections and Male genital infections) | Kansenshogaku Zasshi | JPN01 |
|  | Yuka Asai, Akerke Baibergenova, Maha Dutil, Shannon Humphrey, Peter Hull, Charles Lynde, Yves Poulin, Neil H. Shear, Jerry Tan, John Toole, Catherine Zip | 2015 | Management of acne | CPG Infobase | CAN03 |

**Additional file D: List of excluded studies**

|  | Author | Date | Title | Journal | Reason |
| --- | --- | --- | --- | --- | --- |
|  | Japanese Society of Chemotherapy Committee on guidelines for treatment of anaerobic, infections and Japanese Association for Anaerobic Infection, Research | 2011 | Chapter 2-11. Anaerobic infections (individual fields): otorhinolaryngological infections | J Infect Chemother | Book chapter |
|  | Japanese Society of Chemotherapy Committee on guidelines for treatment of anaerobic, infections and Japanese Association for Anaerobic Infection, Research | 2011 | Chapter 2-1. Anaerobic infections (individual fields): respiratory infections | J Infect Chemother | Book chapter |
|  | Japanese Society of Chemotherapy Committee on guidelines for treatment of anaerobic, infections and Japanese Association of Anaerobic Infection, Research | 2011 | Chapter 2-5-3c. Anaerobic infections (individual fields): skin and soft tissue infections--foot infection | J Infect Chemother | Book chapter |
|  | Japanese Society of Chemotherapy Committee on guidelines for treatment of anaerobic, infections and Japanese Association of Anaerobic Infections, Research | 2011 | Chapter 2-5-3a. Anaerobic infections (individual fields): skin and soft tissue infections | J Infect Chemother | Book chapter |
|  | W. s. National Collaborating Centre for and H. Children's | 2015 | National Institute for Health and Care Excellence: Clinical Guidelines | National Institute for Health and Care Excellence: Clinical Guidelines | Book section |
|  | Lopardo, G., Calmaggi, A., Clara, L., Levy Hara, G., Mykietiuk, A., Pryluka, D., Ruvinsky, S., Vujacich, C., Yahni, D., Bogdanowicz, E., Klein, M., López Furst, M. J., Pensotti, C., Rial, M. J. and Scapellato, P. | 2012 | [Consensus guidelines for the management of upper respiratory tract infections] | Medicina (B Aires) | Consensus document |
|  | Prieto, L., Esteban, M., Salinas, J., Adot, J. M., Arlandis, S., Peri, L. and Cozar, J. M. | 2015 | Consensus document of the Spanish Urological Association on the management of uncomplicated recurrent urinary tract infections | Actas Urol Esp | Consensus document |
|  | Alsubaie, H., Al-Shamrani, A., Alharbi, A. S. and Alhaider, S. | 2015 | Clinical practice guidelines: Approach to cough in children: The official statement endorsed by the Saudi Pediatric Pulmonology Association (SPPA) | Int J Pediatr Adolesc Med | Consensus document |
|  | Sánchez-Hernández, M. C., Montero, J., Rondon, C., Benitez del Castillo, J. M., Velázquez, E., Herreras, J. M., Fernández-Parra, B., Merayo-Lloves, J., Del Cuvillo, A., Vega, F., Valero, A., Panizo, C., Montoro, J., Matheu, V., Lluch-Bernal, M., González, M. L., González, R., Dordal, M. T., Dávila, I., Colás, C., Campo, P., Antón, E. and Navarro, A. | 2015 | Consensus document on allergic conjunctivitis (DECA) | J Investig Allergol Clin Immunol | Consensus document |
|  | Nemirovsky, C., López Furst, M. J., Pryluka, D., De Vedia, L., Scapellato, P., Colque, A., Barcelona, L., Desse, J., Caradonti, M., Varcasia, D., Ipohorski, G., Votta, R., Zylberman, M., Romani, A., Valdez, P., Amalfa, F., Lucero, C., Fernández Lausi, A., Fernández Garces, A., Rodríguez, C., Chattas, A., Farina, J., Clara, L. and Nuccetelli, Y. | 2020 | [Argentine Intersociety Consensus on Urinary Infection 2018-2019 - Part II] | Medicina (B Aires) | Consensus document |
|  | Nemirovsky, C., López Furst, M. J., Pryluka, D., De Vedia, L., Scapellato, P., Colque, A., Barcelona, L., Desse, J., Caradonti, M., Varcasia, D., Ipohorski, G., Votta, R., Zylberman, M., Romani, A., Valdez, P., Penini, M., De Paulis, A., Lucero, C., Sandor, A., Contreras, R., Nannini, E., Gañete, M., Ralli, H., Lopardo, G., Mykietiuk, A. and Aronson, S. | 2020 | [Consenso Argentino Intersociedades de Infección Urinaria 2018-2019 - Parte I] | Medicina (B Aires) | Consensus document |
|  | Federacao Brasileira das Associacoes de Ginecologia e, Obstetricia, Sociedade Brasileira de, Infectologia, Sociedade Brasileira de Medicina de Familia e, Comunidade, Sociedade Brasileira de, Nefrologia and Colegio Brasileiro de, Radiologia | 2011 | Uncomplicated urinary infection in women: diagnosis | Rev Assoc Med Bras (1992) | Diagnostic guideline |
|  |  | 2020 | Urinary Tract Infections in the Primary Care Setting – Investigation | Clinical Practice Guidelines and Protocols in British Columbia | Diagnostic guideline |
|  |  | 2011 | Epp A, Larochelle A | SOGC Urogynaecology Committee, SOGC Family Physicians Advisory Committee. Recurrent urinary tract infection. SOGC Clinical Practice Guideline No. 250, November 2010. J Obstet Gynaecol Can 2010 | Duplicate |
|  |  | 2011 | Canadian clinical practice guidelines for acute and chronic rhinosinusitis | CPG Infobase | Duplicate |
|  | Wächtler, H. and Chenot, J. F. | 2011 | Guidelines for the management of sore throat from the German Society of General Practice and Family Medicine | Hno | Duplicate |
|  |  | 2011 | Guidelines for Antimicrobial Treatment of Acute Uncomplicated Cystitis and Pyelonephritis in Women | Infectious Diseases Society of America | Duplicate |
|  | Yasuda, M., Takahashi, S., Kiyota, H., Ishikawa, K., Takahashi, A., Yamamoto, S., Arakawa, S., Monden, K., Muratani, T., Hamasuna, R., Hayami, H. and Matsumoto, T. | 2011 | Japanese guideline for clinical research of antimicrobial agents on urogenital infections: the first edition | J Infect Chemother | Duplicate |
|  |  | 2012 | IDSA Clinical Practice Guideline for Acute Bacterial Rhinosinusitis in Children and Adults | Infectious Diseases Society of America | Duplicate |
|  |  | 2012 | Clinical Practice Guideline for the Diagnosis and Management of Group A Streptococcal Pharyngitis | Infectious Diseases Society of America | Duplicate |
|  | Randel, A. | 2013 | IDSA Updates Guideline for Managing Group A Streptococcal Pharyngitis | Am Fam Physician | Duplicate |
|  | Yamanaka, N. | 2013 | Clinical practice guidelines for the diagnosis and management of acute otitismedia in children in Japan | International Journal of Antimicrobial Agents | Duplicate |
|  | Wald, E. R., Applegate, K. E., Bordley, C., Darrow, D. H., Glode, M. P., Marcy, S. M., Nelson, C. E., Rosenfeld, R. M., Shaikh, N., Smith, M. J., Williams, P. V. and Weinberg, S. T. | 2013 | Clinical practice guideline for the diagnosis and management of acute bacterial sinusitis in children aged 1 to 18 years | Pediatrics | Duplicate |
|  | Dennis L. Stevens, Alan L. Bisno, Henry F. Chambers, E. Patchen Dellinger, Ellie J. C. Goldstein, Sherwood L. Gorbach, Jan V. Hirschmann, Sheldon L. Kaplan, Jose G. Montoya, and James C. Wade |  | Practice Guidelines for the Diagnosis and Management of Skin and Soft Tissue Infections: 2014 Update by the Infectious Diseases Society of America | Infectious Diseases Society of America | Duplicate |
|  | Ralston, S. L., Lieberthal, A. S. and Meissner, H. C. | 2015 | Clinical Practice Guideline: The Diagnosis, Management, and Prevention of Bronchiolitis. Pediatrics. 2014 | 134(5):e1474-e1502 | Duplicate |
|  |  | 2015 | Adult Sinusitis | American Academy of Otolaryngology - Head and Neck Surgery | Duplicate |
|  | Fujieda, S., Sakashita, M., Tokunaga, T., Okano, M., Haruna, Y., Yoshikawa, M., Kou, N., Asaka, D., Haruna, S., Nakayama, T. and Ishidoya, J. | 2015 | Practice guideline for eosinophilic rhinosinusitis | Nihon Jibiinkoka Gakkai Kaiho | Duplicate |
|  |  | 2016 | Adult sinusitis | HAS Guidelines | Duplicate |
|  |  | 2016 | Acute simple cystitis, cystitis with risk of complication or recurrent cystitis in women | HAS Guidelines | Duplicate |
|  | Jones, Stephanie L. and Farrington, Michele | 2016 | American Academy of Otolaryngology-Head and Neck Surgery Foundation Clinical Practice Guidelines | ORL-Head & Neck Nursing | Duplicate |
|  | Epp, A. and Larochelle, A. | 2017 | N° 250-Infection Récurrente Des Voies Urinaires | J Obstet Gynaecol Can | Duplicate |
|  |  | 2017 | Diagnosis and treatment of respiratory illness in children and adults | National Guideline Clearinghouse (partial archive) | Duplicate |
|  |  | 2018 | Acute Respiratory Illness in Immunocompetent Patients | American College of Radiology | Duplicate |
|  | Kranz, J., Schmidt, S., Lebert, C., Schneidewind, L., Schmiemann, G. and Wagenlehner, F. | 2018 | Clinical practice guideline:Uncomplicated bacterial community acquired urinary tract infection in adults-epidemiology, diagnosis, treatment, and prevention | Journal of Urology | Duplicate |
|  |  | 2019 | Recurrent Uncomplicated Urinary Tract Infections in Women | American Urological Association | Duplicate |
|  |  | 2019 | Respiratory tract infections (self-limiting) – reducing antibiotic prescribing | Best Practice Advocacy Centre New Zealand | Duplicate |
|  |  | 2019 | Asymptomatic Bacteriuria | Infectious Diseases Society of America | Duplicate |
|  |  | 2020 | Adult Sinusitis | American Academy of Family Physicians | Duplicate |
|  |  | 2020 | Recurrent Lower Urinary Tract Infections in Females | American College of Radiology | Duplicate |
|  | File Jr, T. M. | 2011 | Highlights from international clinical practice guidelines for the treatment of acute uncomplicated cystitis and pyelonephritis in women: A 2010 update by the infectious diseases society of America and the European society for microbiology and infectious diseases | Infectious Diseases in Clinical Practice | Editorial |
|  | File, T. M. | 2012 | Highlights from the infectious diseases society of America clinical practice guideline for acute bacterial rhinosinusitis in children and adults | Infectious Diseases in Clinical Practice | Editorial |
|  | Editorial Board of Chinese Journal of Otorhinolaryngology, Head, Neck Surgery Subspecialty Group of, Children, Rhinology, Society of Otorhinolaryngology, Head and Neck Surgery, Chinese Medical Association | 2013 | Suggestion of diagnosis and treatment for pediatric sinusitis | Zhonghua Er Bi Yan Hou Tou Jing Wai Ke Za Zhi | Editorial |
|  | Hauk, L. | 2014 | AAP releases guideline on diagnosis and management of acute bacterial sinusitis in children one to 18 years of age | Am Fam Physician | Editorial |
|  | Politis, P. A. and File, T. M. | 2019 | Highlights of Clinical Practice Guideline for the Management of Asymptomatic Bacteriuria: 2019 Update by the Infectious Diseases Society of America | Infectious Diseases in Clinical Practice | Editorial |
|  | A. K. Goel and M. Kanitkar | 2013 | Streptococcal pharyngitis: an unusual presentation...Bisno AL, Gerber MA, Gwaltney JM Jr, Kaplan EL, Schwartz RH, Infectious Diseases Society of America. Practice guidelines for the diagnosis and management of group A streptococcal pharyngitis. Clin Infect Dis 2002;35:113-25 | Generic | Editorial |
|  | Qaseem, A., Wilt, T. J., Weinberger, S. E., Hanania, N. A., Criner, G., van der Molen, T., Marciniuk, D. D., Denberg, T., Schünemann, H., Wedzicha, W., Macdonald, R. and Shekelle, P. | 2011 | Disease: A clinical practice guideline update from the American college of physicians, American college of chest physicians, American thoracic society, and European respiratory society | Annals of Internal Medicine | Excluded condition |
|  |  | 2011 | Treating cough and cold: Guidance for caregivers of children and youth | Canadian Paediatric Society | Excluded condition |
|  | Wuorela, M., Kouri, T., Laato, M., Lipponen, P., Sammalkorpi, K., Uhari, M., Uusitalo, L. and Vuento, R. | 2011 | [Update on current care guidelines: urinary tract infections] | Duodecim | Excluded condition |
|  | Esposito, S., Bassetti, M., Borre, S., Bouza, E., Dryden, M., Fantoni, M., Gould, I. M., Leoncini, F., Leone, S., Milkovich, G., Nathwani, D., Segreti, J., Sganga, G., Unal, S. and Venditti, M. | 2011 | Diagnosis and management of skin and soft-tissue infections (SSTI): a literature review and consensus statement on behalf of the Italian Society of Infectious Diseases and International Society of Chemotherapy | J Chemother | Excluded condition |
|  | Neumann, I. and Cifuentes, L. | 2012 | Toward trustworthy clinical practice guidelines: the Ministry of Health guideline for management of influenza | Rev Med Chil | Excluded condition |
|  |  | 2013 | Acute otitis externa | Canadian Paediatric Society | Excluded condition |
|  | Lopardo, G., Pensotti, C., Scapellato, P., Caberlotto, O., Calmaggi, A., Clara, L., Klein, M., Levy Hara, G., López Furst, M. J., Mykietiuk, A., Pryluka, D., Rial, M. J., Vujacich, C. and Yahni, D. | 2013 | [Inter-society consensus for the management of respiratory infections: acute bronchitis and chronic obstructive pulmonary disease] | Medicina (B Aires) | Excluded condition |
|  |  | 2014 | Bronchiolitis | American Academy of Family Physicians | Excluded condition |
|  |  | 2014 | Clinical Practice Guideline: The Diagnosis, Management, and Prevention of Bronchiolitis | American Academy of Pediatrics | Excluded condition |
|  | Verstraete, M., Cros, P., Gouin, M., Oillic, H., Bihouée, T., Denoual, H., Barzic, A., Duigou, A. L., Vrignaud, B., Levieux, K., Vabres, N., Fleurence, E., Darviot, E., Cardona, J., Guitteny, M. A., Marot, Y., Picherot, G. and Gras-Le Guen, C. | 2014 | [Update on the management of acute viral bronchiolitis: proposed guidelines of Grand Ouest University Hospitals] | Arch Pediatr | Excluded condition |
|  | Irwin, R. S., French, C. T., Lewis, S. Z., Diekemper, R. L. and Gold, P. M. | 2014 | Overview of the management of cough: CHEST Guideline and Expert Panel Report | Chest | Excluded condition |
|  | Su, S. C. and Chang, A. B. | 2014 | Improving the management of children with bronchiolitis: the updated American Academy of Pediatrics Clinical Practice Guideline | Chest | Excluded condition |
|  |  | 2014 | Fever without focus in children (excluding neonates) | Clinical Practice Guidelines Portal | Excluded condition |
|  | Chiappini, E., Mazzantini, R., Bruzzese, E., Capuano, A., Colombo, M., Cricelli, C., Di Mauro, G., Esposito, S., Festini, F., Guarino, A., Miniello, V. L., Principi, N., Marchisio, P., Rafaniello, C., Rossi, F., Sportiello, L., Tancredi, F., Venturini, E., Galli, L. and de Martino, M. | 2014 | Rational use of antibiotics for the management of children's respiratory tract infections in the ambulatory setting: an evidence-based consensus by the Italian Society of Preventive and Social Pediatrics | Paediatr Respir Rev | Excluded condition |
|  | Ralston, Shawn L., Lieberthal, Allan S., Meissner, H. Cody, Alverson, Brian K., Baley, Jill E., Gadomski, Anne M., Johnson, David W., Light, Michael J., Maraqa, Nizar F., Mendonca, Eneida A., Phelan, Kieran J., Zorc, Joseph J., Stanko-Lopp, Danette, Brown, Mark A., Nathanson, Ian, Rosenblum, Elizabeth, Sayles, Stephen and Hernandez-Cancio, Sinsi | 2014 | Clinical Practice Guideline: The Diagnosis, Management, and Prevention of Bronchiolitis | Pediatrics | Excluded condition |
|  | Hauk, L. | 2015 | AAP releases practice guideline on diagnosis, management, and prevention of bronchiolitis | American Family Physician | Excluded condition |
|  | Prentice, P. | 2015 | American Academy of Otolaryngology: Head and neck surgery foundation clinical practice guideline on acute otitis externa 2014 | Archives of Disease in Childhood: Education and Practice Edition | Excluded condition |
|  | Pollack, C. V., Jr., Amin, A., Ford, W. T., Jr., Finley, R., Kaye, K. S., Nguyen, H. H., Rybak, M. J. and Talan, D. | 2015 | Acute bacterial skin and skin structure infections (ABSSSI): practice guidelines for management and care transitions in the emergency department and hospital | J Emerg Med | Excluded condition |
|  |  | 2015 | Bronchiolitis in children: diagnosis and management | National Institute for Health and Clinical Excellence - Clinical Guidelines | Excluded condition |
|  |  | 2015 | Cough | Scottish Palliative Care Guidelines | Excluded condition |
|  | Osvald, Emma Caffrey, Clarke, Jane R. and Caffrey Osvald, Emma | 2016 | NICE clinical guideline: bronchiolitis in children | Archives of Disease in Childhood -- Education & Practice Edition | Excluded condition |
|  | Yasuda, M., Muratani, T., Ishikawa, K., Kiyota, H., Sakata, H., Shigemura, K., Takahashi, S., Hamasuna, R., Hayami, H., Mikamo, H., Yamamoto, S., Watanabe, T. and Arakawa, S. | 2016 | Japanese guideline for clinical research of antimicrobial agents on urogenital infections: Second edition | J Infect Chemother | Excluded condition |
|  | Kirsch, C. F. E., Bykowski, J., Aulino, J. M., Berger, K. L., Choudhri, A. F., Conley, D. B., Luttrull, M. D., Nunez, D., Jr., Shah, L. M., Sharma, A., Shetty, V. S., Subramaniam, R. M., Symko, S. C. and Cornelius, R. S. | 2017 | ACR Appropriateness Criteria(®) Sinonasal Disease | J Am Coll Radiol | Excluded condition |
|  |  | 2017 | Urethritis | New Zealand Sexual Health Society | Excluded condition |
|  |  | 2018 | Sinusitis - Child | American College of Radiology | Excluded condition |
|  |  | 2018 | Antibiotics after incision and drainage for uncomplicated skin abscesses | BMJ Rapid Recommendations | Excluded condition |
|  |  | 2018 | The use of antiviral drugs for influenza: Guidance for practitioners | Canadian Paediatric Society | Excluded condition |
|  | Kou, M., Hwang, V. and Ramkellawan, N. | 2018 | Bronchiolitis: From Practice Guideline to Clinical Practice | Emerg Med Clin North Am | Excluded condition |
|  | Brubaker, L., Carberry, C., Nardos, R., Carter-Brooks, C. and Lowder, J. L. | 2018 | American Urogynecologic Society Best-Practice Statement: Recurrent Urinary Tract Infection in Adult Women | Female Pelvic Med Reconstr Surg | Excluded condition |
|  |  | 2018 | Classification of cough as a symptom in adults and management algorithms: CHEST guideline and Expert Panel report | National Guideline Clearinghouse (partial archive) | Excluded condition |
|  |  | 2018 | Bronchiolitis in Children | SA Health Paediatric Clinical Practice Guidelines | Excluded condition |
|  |  | 2019 | Recommendations for Prevention and Control of Influenza in Children, 2019–2020 | American Academy of Pediatrics | Excluded condition |
|  |  | 2019 | Recurrent uncomplicated urinary tract infections in women: AUA/CUA/SUFU | Canadian Urological Association | Excluded condition |
|  | O'Brien, S., Borland, M. L., Cotterell, E., Armstrong, D., Babl, F., Bauert, P., Brabyn, C., Garside, L., Haskell, L., Levitt, D., McKay, N., Neutze, J., Schibler, A., Sinn, K., Spencer, J., Stevens, H., Thomas, D., Zhang, M., Oakley, E. and Dalziel, S. R. | 2019 | Australasian bronchiolitis guideline | J Paediatr Child Health | Excluded condition |
|  | Chiappini, E., Ciarcià, M., Bortone, B., Doria, M., Becherucci, P., Marseglia, G. L., Motisi, M. A., de Martino, M., Galli, L., Licari, A., De Masi, S., Lubrano, R., Bettinelli, M., Vicini, C., Felisati, G., Villani, A. and Marchisio, P. | 2019 | Updated Guidelines for the Management of Acute Otitis Media in Children by the Italian Society of Pediatrics: Diagnosis | Pediatr Infect Dis J | Excluded condition |
|  | Bréver, A., Tomat, A. M. and Seghaye, M. C. | 2019 | Update on the management of bronchiolitis | Rev Med Liege | Excluded condition |
|  | Apewokin, S. and Onyishi, N. | 2020 | Influenza-like Illness Definition Pertaining to Clinical Practice Guidelines on the Diagnosis, Treatment, Chemoprophylaxis, and Institutional Outbreak Management of Seasonal Influenza | Clin Infect Dis | Excluded condition |
|  | Apewokin, S. | 2020 | Influenza-like illness definition pertaining to clinical practice guidelines on the diagnosis, treatment, chemoprophylaxis, and institutional outbreak management of seasonal influenza | Clinical Infectious Diseases | Excluded condition |
|  |  | 2021 | Secondary bacterial infection of eczema and other common skin conditions: antimicrobial prescribing | National Institute for Health and Clinical Excellence - Clinical Guidelines | Excluded condition |
|  | A. Sucher, A. Do and M. Negash | 2019 | Latest clinical practice guidelines for seasonal influenza | Embase | Excluded condition |
|  | Force, U. S. Preventive Services Task | 2010 | Screening for asymptomatic bacteriuria in adults: reaffirmation recommendation statement | Am Fam Physician | Excluded date |
|  | Simó Nebot, M., Claret Teruel, G., Luaces Cubells, C., Estrada Sabadell, M. D. and Pou Fernández, J. | 2010 | Acute bronchiolitis clinical practice guideline: Recommendations for clinical practice | Anales de Pediatria | Excluded date |
|  |  | 2010 | Update on current care guidelines: Acute otitis media | Duodecim | Excluded date |
|  | Ranki, A., Hyry, H., Klimenko, T., Laukkala, T., Majamaa, H., Mäkelä, M., Syrjänen, J. and Vuopio, J. | 2010 | [Update on current care guidelines. Bacterial skin infections] | Duodecim | Excluded date |
|  | Del Pilar Velázquez, M., Romero Nava, L. E., López de Avalos, D. R., Quiroz Garza, G., Solano Sánchez, R., Gorbea Chávez, V., Iris de la Cruz, S., Villagrana Zesatti, R., Arredondo García, J. L. and Figueroa Damián, R. | 2010 | [Clinical practice guidelines. Recurrent infection of the urinary tract in women. Colegio Mexicano de Especialistas en Ginecología y Obstetricia] | Ginecol Obstet Mex | Excluded date |
|  | Epp, A. and Larochelle, A. | 2010 | Recurrent urinary tract infection | J Obstet Gynaecol Can | Excluded date |
|  | Gibson, P. G., Chang, A. B., Glasgow, N. J., Holmes, P. W., Katelaris, P., Kemp, A. S., Landau, L. I., Mazzone, S., Newcombe, P., Van Asperen, P. and Vertigan, A. E. | 2010 | CICADA: Cough in Children and Adults: Diagnosis and Assessment. Australian cough guidelines summary statement | Med J Aust | Excluded date |
|  |  | 2010 | CICADA: Cough in Children and Adults: Diagnosis and Assessment. Australian Cough Guidelines summary statement | MJA Clinical Guidelines | Excluded date |
|  |  | 2010 | Lower urinary tract symptoms in men: management | National Institute for Health and Clinical Excellence - Clinical Guidelines | Excluded date |
|  | Kardos, P., Berck, H., Fuchs, K. H., Gillissen, A., Klimek, L., Morr, H., Pfeiffer-Kascha, D., Schultze-Werninghaus, G., Sitter, H., Voshaar, T. and Worth, H. | 2010 | Guidelines of the German Respiratory Society for diagnosis and treatment of adults suffering from acute or chronic cough | Pneumologie | Excluded date |
|  | Green, R. J., Zar, H. J., Jeena, P. M., Madhi, S. A. and Lewis, H. | 2010 | South African guideline for the diagnosis, management and prevention of acute viral bronchiolitis in children | S Afr Med J | Excluded date |
|  |  | 2010 | Croup - assessment and management | The Royal Australian College of General Practitioners | Excluded date |
|  |  | 2010 | AAO-HNS clinical practice guidelines | Update: Society of Otorhinolaryngology & Head-Neck Nurses | Excluded date |
|  |  | 2011 | Lower urinary tract symptoms. Current management in older men | Clinical Practice Guidelines Portal | Excluded population |
|  | Chidiac, C. | 2011 | [Systemic antibiotherapy for the treatment of lower respiratory tract infections. Community acquired pneumonia, acute exacerbation of obstructive chronic bronchitis] | Med Mal Infect | Excluded population |
|  |  | 2015 | Urological Infections | European Association of Urology | Excluded population |
|  |  | 2015 | Treatment of Non-neurogenic Male LUTS | European Association of Urology | Excluded population |
|  |  | 2018 | Urological Infections | European Association of Urology | Excluded population |
|  |  | 2018 | Management of Non-neurogenic Male LUTS | European Association of Urology | Excluded population |
|  | Piñeiro Pérez, R., Cilleruelo Ortega, M. J., Ares Álvarez, J., Baquero-Artigao, F., Silva Rico, J. C., Velasco Zúñiga, R., Martínez Campos, L., Carazo Gallego, B., Conejo Fernández, A. J. and Calvo, C. | 2019 | [Recommendations on the diagnosis and treatment of urinary tract infection] | An Pediatr (Barc) | Excluded population |
|  |  | 2019 | Management of Non-neurogenic Male LUTS | European Association of Urology | Excluded population |
|  |  | 2019 | Urological Infections | European Association of Urology | Excluded population |
|  | Sunderkötter, C., Becker, K., Eckmann, C., Graninger, W., Kujath, P. and Schöfer, H. | 2019 | S2k guidelines for skin and soft tissue infections Excerpts from the S2k guidelines for "calculated initial parenteral treatment of bacterial infections in adults - update 2018" | J Dtsch Dermatol Ges | Excluded population |
|  |  | 2013 | Pharyngitis | University of Michigan Health System | Local guideline |
|  |  | 2014 | Acute croup in children | Clinical Practice Guidelines Portal | Local guideline |
|  |  | 2014 | Otitis Media | University of Michigan Health System | Local guideline |
|  |  | 2015 | Croup | Accelerating Change Transformation Team | Local guideline |
|  |  | 2016 | Urinary Tract Infection | University of Michigan Health System | Local guideline |
|  | Mospan, G. and Hulisz, D. | 2017 | Treating skin and soft tissue infections | Jaapa | Local guideline |
|  |  | 2018 | Conjunctivitis (bacterial) | College of Optometrists | Local guideline |
|  |  | 2018 | Acute Rhinosinusitis in Adults | University of Michigan Health System | Local guideline |
|  |  | 2020 | Conjunctivitis (viral, non-herpetic) | College of Optometrists | Local guideline |
|  |  | 2016 | Management of fever in children | HAS Guidelines | Not retrieved |
|  |  | 2016 | Acute nasopharyngitis and acute strep throat in children | HAS Guidelines | Not retrieved |
|  |  | 2016 | Acute nasopharyngitis and acute strep throat in adults | HAS Guidelines | Not retrieved |
|  | Mace, S. E., Gemme, S. R., Valente, J. H., Eskin, B., Bakes, K., Brecher, D. and Brown, M. D. | 2016 | Clinical Policy for Well-Appearing Infants and Children Younger Than 2 Years of Age Presenting to the Emergency Department With Fever | Ann Emerg Med | Policy document |
|  | Dibildox-Martinez, J., Mayorga Butron, J. L., Macías Fernández, L. A., Casiano, R. R., Carrau, R. L., Javer, A. R. and Gordon, B. R. | 2012 | Pan-american clinical guideline on rhinosinusitis | Otolaryngology - Head and Neck Surgery (United States) | Poster |
|  |  | 2015 | Prevention of Acute Exacerbations of COPD | American College of Chest Physicians | Preventive guideline |
|  | Marchisio, P., Bortone, B., Ciarcià, M., Motisi, M. A., Torretta, S., Castelli Gattinara, G., Picca, M., Di Mauro, G., Bonino, M., Mansi, N., Varricchio, A., Marseglia, G. L., Cardinale, F., Villani, A. and Chiappini, E. | 2019 | Updated Guidelines for the Management of Acute Otitis Media in Children by the Italian Society of Pediatrics: Prevention | Pediatr Infect Dis J | Preventive guideline |
|  | Committee On Infectious, Diseases | 2019 | Recommendations for Prevention and Control of Influenza in Children, 2019-2020 | Pediatrics | Preventive guideline |
|  |  | 2012 | New practice guideline for rhinosinusitis | Critical Care Alert | Report |
|  | Elliott, William T. | 2012 | New practice guideline for rhinosinusitis | Primary Care Reports | Report |
|  | Ogletree, R. L., Jr. | 2013 | About group A streptococcal pharyngitis guidelines | J Miss State Med Assoc | Report |
|  | Riechelmann, H., Giotakis, A. and Kral, F. | 2013 | [Acute rhinosinusitis in adults--EPOS 2012 Part II] | Laryngorhinootologie | Report |
|  | Hersh, A. L., Jackson, M. A. and Hicks, L. A. | 2013 | Principles of judicious antibiotic prescribing for upper respiratory tract infections in pediatrics | Pediatrics | Report |
|  | Harrison, Melody | 2014 | Otitis Media--Red Flags for Management: American Academy of Otolaryngology | Head and Neck Surgery 2013 Clinical Practice Guideline for Tympanostomy Tube Insertion | Report |
|  | Rosenfeld, R. M., Schwartz, S. R., Cannon, C. R., Roland, P. S., Simon, G. R., Kumar, K. A., Huang, W. W., Haskell, H. W. and Robertson, P. J. | 2014 | Clinical practice guideline: Acute otitis externa executive summary | Otolaryngology - Head and Neck Surgery (United States) | Report |
|  | de Jongh, E. and Opstelten, W. | 2015 | Revision of the Dutch College of General Practitioners practice guideline 'Acute sore throat' | Ned Tijdschr Geneeskd | Report |
|  |  | 2015 | Sinusitis: Scenario: Acute sinusitis | NICE Clinical Knowledge Summaries | Report |
|  |  | 2015 | Sinusitis: How should I diagnose acute sinusitis? | NICE Clinical Knowledge Summaries | Report |
|  |  | 2015 | Sinusitis: Differential diagnosis | NICE Clinical Knowledge Summaries | Report |
|  |  | 2016 | Appropriate Antibiotic Use for Acute Respiratory Tract Infection in Adults: Advice for High-Value Care From the American College of Physicians and the Centers for Disease Control and Prevention | American College of Physicians | Report |
|  | Harris, A. M., Hicks, L. A. and Qaseem, A. | 2016 | Appropriate Antibiotic Use for Acute Respiratory Tract Infection in Adults: Advice for High-Value Care From the American College of Physicians and the Centers for Disease Control and Prevention | Ann Intern Med | Report |
|  |  | 2016 | Sore throat - acute | NICE Clinical Knowledge Summaries | Report |
|  |  | 2016 | Otitis media - acute: Scenario: Recurrent acute otitis media | NICE Clinical Knowledge Summaries | Report |
|  |  | 2016 | Otitis media - acute: Scenario: Persistent acute otitis media - treatment failure | NICE Clinical Knowledge Summaries | Report |
|  |  | 2016 | Otitis media - acute: Scenario: Acute otitis media - initial presentation | NICE Clinical Knowledge Summaries | Report |
|  |  | 2016 | Otitis media - acute: Prescribing paracetamol and ibuprofen | NICE Clinical Knowledge Summaries | Report |
|  |  | 2016 | Otitis media - acute: How should I make a diagnosis of acute otitis media? | NICE Clinical Knowledge Summaries | Report |
|  |  | 2016 | Otitis media - acute: Erythromycin | NICE Clinical Knowledge Summaries | Report |
|  |  | 2016 | Otitis media - acute: Co-amoxiclav | NICE Clinical Knowledge Summaries | Report |
|  |  | 2016 | Otitis media - acute: Clarithromycin | NICE Clinical Knowledge Summaries | Report |
|  |  | 2016 | Otitis media - acute: Amoxicillin | NICE Clinical Knowledge Summaries | Report |
|  |  | 2016 | Otitis media - acute | NICE Clinical Knowledge Summaries | Report |
|  |  | 2016 | Otitis externa: What else might it be? | NICE Clinical Knowledge Summaries | Report |
|  |  | 2017 | Pharmacologic and Nonpharmacologic Treatment for Acute Cough Associated With the Common Cold | American College of Chest Physicians | Report |
|  | Velez, Roseann, Richmond, Elizabeth and Dudley-Brown, Sharon | 2017 | Antibiogram, Clinical Practice Guidelines, and Treatment of Urinary Tract Infection | Journal for Nurse Practitioners | Report |
|  | Eizenga, W. E. and Opstelten, W. | 2017 | Revision of the Dutch College of General Practitioners practice guideline 'Children with fever' | Ned Tijdschr Geneeskd | Report |
|  |  | 2017 | Otitis media - acute: What else might it be? | NICE Clinical Knowledge Summaries | Report |
|  |  | 2017 | Otitis externa: Scenario: Localized otitis externa | NICE Clinical Knowledge Summaries | Report |
|  |  | 2017 | Otitis externa: Scenario: Acute diffuse otitis externa | NICE Clinical Knowledge Summaries | Report |
|  |  | 2017 | LUTS in men: What investigations should I arrange for a man presenting with lower urinary tract symptoms? | NICE Clinical Knowledge Summaries | Report |
|  |  | 2017 | LUTS in men: Scenario: Voiding symptoms | NICE Clinical Knowledge Summaries | Report |
|  |  | 2017 | LUTS in men: How should I assess a man with lower urinary tract symptoms? | NICE Clinical Knowledge Summaries | Report |
|  |  | 2017 | LUTS in men | NICE Clinical Knowledge Summaries | Report |
|  |  | 2017 | Feverish children - risk assessment | NICE Clinical Knowledge Summaries | Report |
|  |  | 2017 | Conjunctivitis - infective: What else might it be? | NICE Clinical Knowledge Summaries | Report |
|  |  | 2017 | Conjunctivitis - infective: What are the clinical features of infective conjunctivitis? | NICE Clinical Knowledge Summaries | Report |
|  |  | 2017 | Conjunctivitis - infective: Topical fusidic acid | NICE Clinical Knowledge Summaries | Report |
|  |  | 2017 | Conjunctivitis - infective: Topical chloramphenicol | NICE Clinical Knowledge Summaries | Report |
|  |  | 2017 | Conjunctivitis - infective: Scenario: Who should I refer to ophthalmology? | NICE Clinical Knowledge Summaries | Report |
|  |  | 2017 | Conjunctivitis - infective: Scenario: Management in primary care | NICE Clinical Knowledge Summaries | Report |
|  |  | 2017 | Conjunctivitis - infective: How should I assess a person with suspected infective conjunctivitis? | NICE Clinical Knowledge Summaries | Report |
|  |  | 2017 | Conjunctivitis - infective | NICE Clinical Knowledge Summaries | Report |
|  |  | 2017 | Chest infections - adult: Scenario: Acute bronchitis | NICE Clinical Knowledge Summaries | Report |
|  |  | 2017 | Chest infections - adult: How should I assess an adult with a suspected chest infection? | NICE Clinical Knowledge Summaries | Report |
|  |  | 2017 | Chest infections - adult | NICE Clinical Knowledge Summaries | Report |
|  |  | 2018 | Classification of Cough as a Symptom in Adults and Management Algorithms | American College of Chest Physicians | Report |
|  | Vermandere, M., Aertgeerts, B., Agoritsas, T., Liu, C., Burgers, J., Merglen, A., Okwen, P. M., Lytvyn, L., Chua, S., Vandvik, P. O., Guyatt, G. H., Beltran-Arroyave, C., Lavergne, V., Speeckaert, R., Steen, F. E., Arteaga, V., Sender, R., McLeod, S., Sun, X., Wang, W. and Siemieniuk, R. A. C. | 2018 | Antibiotics after incision and drainage for uncomplicated skin abscesses: A clinical practice guideline | BMJ (Online) | Report |
|  | Omoruyi, E. A. | 2018 | Practice Guideline: Approach to the Child with Rhinorrhea | J Pediatr Health Care | Report |
|  |  | 2018 | Cough: Scenario: Management | NICE Clinical Knowledge Summaries | Report |
|  |  | 2018 | Cough: Proton pump inhibitors | NICE Clinical Knowledge Summaries | Report |
|  |  | 2018 | Cough: Nasal corticosteroids | NICE Clinical Knowledge Summaries | Report |
|  |  | 2018 | Cough: How should I assess a person with cough? | NICE Clinical Knowledge Summaries | Report |
|  |  | 2018 | Cough: How do I diagnose the cause of cough? | NICE Clinical Knowledge Summaries | Report |
|  |  | 2018 | Cough: Beta-2 agonists | NICE Clinical Knowledge Summaries | Report |
|  |  | 2018 | Cough - acute with chest signs in children | NICE Clinical Knowledge Summaries | Report |
|  |  | 2018 | Cough | NICE Clinical Knowledge Summaries | Report |
|  |  | 2018 | Cellulitis - acute: What else might it be? | NICE Clinical Knowledge Summaries | Report |
|  |  | 2018 | Cellulitis - acute: Scenario: Management of acute cellulitis | NICE Clinical Knowledge Summaries | Report |
|  |  | 2018 | Cellulitis - acute: Metronidazole | NICE Clinical Knowledge Summaries | Report |
|  |  | 2018 | Cellulitis - acute: How should I diagnose cellulitis? | NICE Clinical Knowledge Summaries | Report |
|  |  | 2018 | Cellulitis - acute: Flucloxacillin | NICE Clinical Knowledge Summaries | Report |
|  |  | 2018 | Cellulitis - acute: Erythromycin | NICE Clinical Knowledge Summaries | Report |
|  |  | 2018 | Cellulitis - acute: Doxycycline | NICE Clinical Knowledge Summaries | Report |
|  |  | 2018 | Cellulitis - acute: Co-amoxiclav | NICE Clinical Knowledge Summaries | Report |
|  |  | 2018 | Cellulitis - acute: Clarithromycin | NICE Clinical Knowledge Summaries | Report |
|  |  | 2018 | Cellulitis - acute: Analgesia | NICE Clinical Knowledge Summaries | Report |
|  |  | 2018 | Cellulitis - acute: Amoxicillin | NICE Clinical Knowledge Summaries | Report |
|  | Mazurek, H., Bręborowicz, A., Doniec, Z., Emeryk, A., Krenke, K., Kulus, M. and Zielnik-Jurkiewicz, B. | 2019 | Acute subglottic laryngitis. Etiology, epidemiology, pathogenesis and clinical picture | Adv Respir Med | Report |
|  | Alvarez-Moreno, C. A. | 2019 | Clinical practice guidelines: An opportunity to provide health care with excellence and the New Colombian guidelines for the diagnosis and management of Skin and Soft tissue infections (SSTIs) | Infectio | Report |
|  |  | 2019 | Urinary tract infection (lower) - women | NICE Clinical Knowledge Summaries | Report |
|  |  | 2019 | Urinary tract infection (lower) - men | NICE Clinical Knowledge Summaries | Report |
|  |  | 2019 | Influenza - seasonal: Zanamivir | NICE Clinical Knowledge Summaries | Report |
|  |  | 2019 | Influenza - seasonal: What else might cause symptoms of influenza-like illness? | NICE Clinical Knowledge Summaries | Report |
|  |  | 2019 | Influenza - seasonal: Scenario: Treatment of influenza | NICE Clinical Knowledge Summaries | Report |
|  |  | 2019 | Influenza - seasonal: Scenario: Post-exposure prophylaxis of influenza | NICE Clinical Knowledge Summaries | Report |
|  |  | 2019 | Impetigo: What else might it be? | NICE Clinical Knowledge Summaries | Report |
|  |  | 2019 | Impetigo: What are the clinical features? | NICE Clinical Knowledge Summaries | Report |
|  |  | 2019 | Impetigo: How should I assess a person with suspected impetigo? | NICE Clinical Knowledge Summaries | Report |
|  |  | 2019 | Impetigo | NICE Clinical Knowledge Summaries | Report |
|  |  | 2019 | Croup | NICE Clinical Knowledge Summaries | Report |
|  |  | 2019 | Common cold: What else might it be? | NICE Clinical Knowledge Summaries | Report |
|  |  | 2019 | Common cold: Scenario: Management | NICE Clinical Knowledge Summaries | Report |
|  |  | 2019 | Common cold: Paracetamol | NICE Clinical Knowledge Summaries | Report |
|  |  | 2019 | Common cold: Ibuprofen | NICE Clinical Knowledge Summaries | Report |
|  |  | 2019 | Common cold: How should I diagnose the common cold in younger children and infants? | NICE Clinical Knowledge Summaries | Report |
|  |  | 2019 | Common cold: How should I diagnose the common cold in adults and older children? | NICE Clinical Knowledge Summaries | Report |
|  |  | 2019 | Common cold | NICE Clinical Knowledge Summaries | Report |
|  |  | 2019 | Chronic obstructive pulmonary disease: Scenario: Acute exacerbation of chronic obstructive pulmonary disease | NICE Clinical Knowledge Summaries | Report |
|  |  | 2019 | Boils, carbuncles, and staphylococcal carriage: What else might it be? | NICE Clinical Knowledge Summaries | Report |
|  |  | 2019 | Boils, carbuncles, and staphylococcal carriage: Scenario: Staphylococcal carriage | NICE Clinical Knowledge Summaries | Report |
|  |  | 2019 | Boils, carbuncles, and staphylococcal carriage: Scenario: Boils and carbuncles | NICE Clinical Knowledge Summaries | Report |
|  |  | 2019 | Boils, carbuncles, and staphylococcal carriage: How do I know my patient has a boil? | NICE Clinical Knowledge Summaries | Report |
|  |  | 2019 | Boils, carbuncles, and staphylococcal carriage: Clinical presentation of a carbuncle | NICE Clinical Knowledge Summaries | Report |
|  |  | 2019 | Boils, carbuncles, and staphylococcal carriage | NICE Clinical Knowledge Summaries | Report |
|  |  | 2019 | Acne vulgaris: What are the clinical features of acne vulgaris? | NICE Clinical Knowledge Summaries | Report |
|  | Helbig, S. and Mandraka, F. | 2020 | Clinical guideline: Uncomplicated urinary tract infection. What is new? | Gynakologische Praxis | Report |
|  |  | 2020 | Red eye: Scenario: Management of red eye | NICE Clinical Knowledge Summaries | Report |
|  |  | 2020 | Paronychia - acute: Erythromycin | NICE Clinical Knowledge Summaries | Report |
|  |  | 2020 | Paronychia - acute: Clarithromycin | NICE Clinical Knowledge Summaries | Report |
|  |  | 2020 | Paronychia - acute | NICE Clinical Knowledge Summaries | Report |
|  |  | 2020 | Otitis externa | NICE Clinical Knowledge Summaries | Report |
|  |  | 2020 | Influenza - seasonal: Oseltamivir | NICE Clinical Knowledge Summaries | Report |
|  |  | 2020 | Influenza - seasonal: How should I diagnose seasonal influenza? | NICE Clinical Knowledge Summaries | Report |
|  |  | 2020 | Influenza - seasonal | NICE Clinical Knowledge Summaries | Report |
|  |  | 2020 | Cellulitis - acute | NICE Clinical Knowledge Summaries | Report |
|  | World Health Organization | 2012 | WHO Guidelines Approved by the Guidelines Review Committee | WHO | Report |
|  | Boulet, L. P., Turmel, J. and Irwin, R. S. | 2017 | Cough in the Athlete: CHEST Guideline and Expert Panel Report | Chest | Report Expert Pane tee |
|  | van Asselt, K. M., Prins, J. M., van der Weele, G. M., Knottnerus, B. J., van Pinxteren, B. and Geerlings, S. E. | 2013 | Unambiguous practice guidelines on urinary tract infections in primary and secondary care | Ned Tijdschr Geneeskd | Review |
|  | Verlee, L., Verheij, T. J., Hopstaken, R. M., Prins, J. M., Salomé, P. L. and Bindels, P. J. | 2012 | Summary of NHG practice guideline 'Acute cough' | Ned Tijdschr Geneeskd | Summary report |
|  | Comite Nacional de, Neumonologia, Comite Nacional de, Infectologia and Comite Nacional de Medicina, Interna | 2015 | [Recommendations for the management of acute lower respiratory infections in children under 2 years of age. Executive summary] | Arch Argent Pediatr | Summary report |
|  | Jäckel, M. C. | 2016 | [The new guideline "Treatment of Inflammatory Diseases of the Palatine Tonsils - Tonsillitis" : Impact in clinics and surgeries] | Hno | Surgical intervention guideline |
|  | Subcommittee of Clinical Practice Guideline for, Diagnosis and Management of Acute Otitis Media in, Children | 2012 | Clinical practice guidelines for the diagnosis and management of acute otitis media (AOM) in children in Japan | Auris Nasus Larynx | Update available |
|  | Kitamura, K., Iino, Y., Kamide, Y., Kudo, F., Nakayama, T., Suzuki, K., Taiji, H., Takahashi, H., Yamanaka, N. and Uno, Y. | 2015 | Clinical practice guidelines for the diagnosis and management of acute otitis media (AOM) in children in Japan - 2013 update | Auris Nasus Larynx | Update available |
|  | Institut national d'excellence en santé et en services sociaux** | 2017 | Antibiotics - acute bronchitis | https://joulecma.ca/cpg/homepage | Consensus document |
|  | Institut national d'excellence en santé et en services sociaux** | 2017 | Sinusitis | https://joulecma.ca/cpg/homepage | Consensus document |
|  | World Health Organization** | 2012 | WHO Guidelines Approved by the Guidelines Review Committee | https://www.who.int/publications/who-guidelines | Duplicate |
|  | The 2017 Update of the German Clinical Guideline on Epidemiology, Diagnostics, Therapy, Prevention, and Management of Uncomplicated Urinary Tract Infections in Adult Patients** | 2018 | Association of Scientific Medical Societies (AWMF) | https://www.awmf.org/leitlinien/leitlinien-suche.html | Duplicate |

** *From guideline websites*

**Additional File E: Quality Assessment by AGREE II of 82 Evidence-based Guidelines**

|  | | | | | | |
| --- | --- | --- | --- | --- | --- | --- |
| Guideline I.D. | Scope and purpose (%) | Stakeholders involvement (%) | Rigour of development (%) | Clarity of presentation (%) | Applicability (%) | Editorial independence (%) |
| AUS01 | 63.9 | 22.2 | 11.5 | 58.3 | 20.8 | 8.3 |
| AUS02 | 58.3 | 19.4 | 12.5 | 63.9 | 41.7 | 4.2 |
| AUS03 | 86.1 | 44.4 | 41.7 | 86.1 | 35.4 | 58.3 |
| BEL01 | 83.3 | 55.6 | 34.4 | 75.0 | 37.5 | 33.3 |
| BEL02 | 83.3 | 55.6 | 34.4 | 75.0 | 35.4 | 33.3 |
| CAN02 | 86.1 | 69.4 | 84.4 | 91.7 | 54.2 | 70.8 |
| CAN03 | 94.4 | 91.7 | 85.4 | 88.9 | 85.4 | 83.3 |
| CAN04 | 80.6 | 61.1 | 83.3 | 83.3 | 66.7 | 87.5 |
| CAN05 | 80.6 | 52.8 | 18.8 | 50.0 | 37.5 | 41.7 |
| CHI01 | 72.2 | 69.4 | 76.0 | 55.6 | 54.2 | 70.8 |
| COL01 | 83.3 | 86.1 | 83.3 | 83.3 | 70.8 | 62.5 |
| COL02 | 52.8 | 41.7 | 37.5 | 58.3 | 54.2 | 25.0 |
| DEN01 | 94.4 | 100.0 | 95.8 | 88.9 | 75.0 | 100.0 |
| DEN02 | 75.0 | 61.1 | 65.6 | 88.9 | 43.8 | 45.8 |
| FIN01 | 66.7 | 55.6 | 75.0 | 69.4 | 45.8 | 4.2 |
| FIN02 | 80.6 | 77.8 | 39.6 | 80.6 | 50.0 | 87.5 |
| FIN03 | 75.0 | 77.8 | 39.6 | 80.6 | 50.0 | 87.5 |
| FIN04 | 75.0 | 77.8 | 39.6 | 80.6 | 50.0 | 87.5 |
| FIN05 | 80.6 | 77.8 | 38.5 | 83.3 | 68.8 | 87.5 |
| FIN06 | 75.0 | 77.8 | 38.5 | 80.6 | 50.0 | 87.5 |
| FRA01 | 61.1 | 63.9 | 58.3 | 66.7 | 47.9 | 41.7 |
| FRA02 | 69.4 | 58.3 | 71.9 | 75.0 | 52.1 | 66.7 |
| FRA03 | 44.4 | 16.7 | 37.5 | 63.9 | 64.6 | 33.3 |
| GER01 | 77.8 | 72.2 | 74.0 | 63.9 | 43.8 | 79.2 |
| GER02 | 38.9 | 36.1 | 18.8 | 63.9 | 25.0 | 4.2 |
| GER03 | 88.9 | 83.3 | 71.9 | 83.3 | 62.5 | 83.3 |
| GER04 | 97.2 | 94.4 | 92.7 | 88.9 | 66.7 | 100.0 |
| GER05 | 83.3 | 91.7 | 87.5 | 94.4 | 79.2 | 100.0 |
| GER07 | 91.7 | 86.1 | 86.5 | 88.9 | 68.8 | 70.8 |
| GER08 | 94.4 | 83.3 | 89.6 | 88.9 | 68.8 | 62.5 |
| INT01 | 86.1 | 66.7 | 76.0 | 83.3 | 56.3 | 54.2 |
| INT02 | 86.1 | 66.7 | 79.2 | 88.9 | 50.0 | 54.2 |
| INT03 | 91.7 | 88.9 | 76.0 | 83.3 | 62.5 | 70.8 |
| INT04 | 86.1 | 75.0 | 77.1 | 77.8 | 66.7 | 75.0 |
| INT05 | 100.0 | 86.1 | 93.8 | 91.7 | 87.5 | 91.7 |
| JPN01 | 80.6 | 47.2 | 69.8 | 86.1 | 62.5 | 41.7 |
| JPN02 | 88.9 | 50.0 | 72.9 | 86.1 | 68.8 | 41.7 |
| JPN03 | 100.0 | 91.7 | 97.9 | 100.0 | 85.4 | 95.8 |
| KOR01 | 91.7 | 94.4 | 87.5 | 77.8 | 56.3 | 29.2 |
| KOR02 | 88.9 | 77.8 | 85.4 | 72.2 | 62.5 | 83.3 |
| KOR04 | 83.3 | 75.0 | 77.1 | 77.8 | 56.3 | 87.5 |
| KOR05 | 91.7 | 80.6 | 89.6 | 83.3 | 66.7 | 87.5 |
| KOR06 | 86.1 | 72.2 | 77.1 | 80.6 | 56.3 | 87.5 |
| MAL01 | 88.9 | 86.1 | 91.7 | 88.9 | 83.3 | 91.7 |
| NED01 | 83.3 | 44.4 | 53.1 | 83.3 | 68.8 | 45.8 |
| NED03 | 80.6 | 61.1 | 62.5 | 83.3 | 60.4 | 70.8 |
| NED04 | 77.8 | 52.8 | 61.5 | 88.9 | 58.3 | 66.7 |
| NED05 | 77.8 | 52.8 | 60.4 | 83.3 | 56.3 | 66.7 |
| NED06 | 75.0 | 52.8 | 58.3 | 83.3 | 58.3 | 66.7 |
| NED07 | 75.0 | 52.8 | 59.4 | 83.3 | 56.3 | 62.5 |
| NED08 | 75.0 | 52.8 | 60.4 | 83.3 | 58.3 | 62.5 |
| NICE02 | 97.2 | 88.9 | 99.0 | 97.2 | 89.6 | 66.7 |
| NICE03 | 100.0 | 83.3 | 97.9 | 97.2 | 91.7 | 66.7 |
| NICE04 | 97.2 | 88.9 | 93.8 | 97.2 | 85.4 | 83.3 |
| NICE05 | 100.0 | 88.9 | 97.9 | 100.0 | 91.7 | 62.5 |
| NICE06 | 100.0 | 83.3 | 100.0 | 100.0 | 91.7 | 66.7 |
| NICE07 | 97.2 | 83.3 | 97.9 | 100.0 | 89.6 | 58.3 |
| NICE08 | 100.0 | 83.3 | 95.8 | 100.0 | 89.6 | 62.5 |
| NICE09 | 100.0 | 83.3 | 94.8 | 100.0 | 91.7 | 62.5 |
| NICE10 | 97.2 | 86.1 | 92.7 | 97.2 | 81.3 | 62.5 |
| NICE11 | 94.4 | 88.9 | 91.7 | 97.2 | 83.3 | 58.3 |
| QAT01 | 94.4 | 86.1 | 79.2 | 91.7 | 81.3 | 87.5 |
| QAT02 | 94.4 | 86.1 | 81.3 | 88.9 | 81.3 | 87.5 |
| RUS01 | 86.1 | 86.1 | 82.3 | 88.9 | 66.7 | 70.8 |
| SCT03 | 100.0 | 94.4 | 96.9 | 94.4 | 79.2 | 91.7 |
| SPN01 | 77.8 | 61.1 | 69.8 | 80.6 | 47.9 | 70.8 |
| USA01 | 72.2 | 50.0 | 66.7 | 61.1 | 29.2 | 66.7 |
| USA02 | 80.6 | 47.2 | 68.8 | 75.0 | 22.9 | 75.0 |
| USA03 | 83.3 | 77.8 | 82.3 | 86.1 | 70.8 | 83.3 |
| USA04 | 88.9 | 86.1 | 88.5 | 86.1 | 70.8 | 87.5 |
| USA05 | 94.4 | 61.1 | 86.5 | 88.9 | 62.5 | 100.0 |
| USA06 | 80.6 | 80.6 | 81.3 | 83.3 | 64.6 | 87.5 |
| USA07 | 100.0 | 88.9 | 92.7 | 88.9 | 85.4 | 91.7 |
| USA08 | 88.9 | 83.3 | 85.4 | 88.9 | 72.9 | 83.3 |
| USA10 | 100.0 | 91.7 | 93.8 | 94.4 | 70.8 | 100.0 |
| USA11 | 83.3 | 61.1 | 75.0 | 75.0 | 56.3 | 79.2 |
| USA12 | 88.9 | 91.7 | 76.0 | 80.6 | 56.3 | 95.8 |
| USA15 | 100.0 | 55.6 | 91.7 | 94.4 | 87.5 | 83.3 |
| USA17 | 100.0 | 75.0 | 92.7 | 91.7 | 81.3 | 83.3 |
| USA20 | 100.0 | 66.7 | 93.8 | 97.2 | 87.5 | 87.5 |
| USA21 | 100.0 | 77.8 | 92.7 | 91.7 | 91.7 | 91.7 |
| WHO1 | 100.0 | 100.0 | 99.0 | 88.9 | 91.7 | 91.7 |
| Median (Range) | 86.1 (38.9-100) | 77.8 (16.7-100) | 79.2(11.5-100) | 84.7 (50.0-100) | 64.6 (20.8-100) | 70.8 (4.2-100) |

**Additional file F: The reported duration of infections reported by only one guideline**


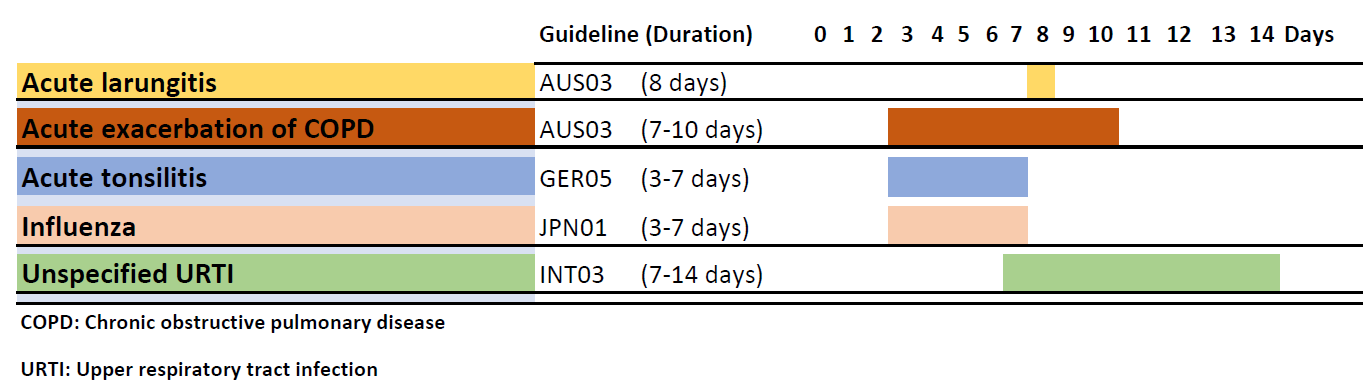


**Additional file G: Antibiotic Stewardship recommendations by guidelines for eligible infections***

| **Condition** | N^†^ | Delayed prescribing | Shared decision making | | Decision aids |
| --- | --- | --- | --- | --- | --- |
| **Respiratory system** | | | | | |
| Acute bronchitis | 12 | 2 (16.6)  [DEN02, NICE04] | 2 (16.6)  [AUS03, NED01] | 3 (25)  [AUS03, NED01, NICE04] | |
| Acute laryngitis | 2 | x | x | x | |
| Acute otitis media | 12 | 11 (91.6)  [AUS01, AUS03, DEN01, DEN02, FIN02, GER07, JPN03, KOR01, NED05, NICE06, USA17] | 5 (41.7)  [AUS03, GER07, JPN03, NED05, USA17] | 3 (25)  [AUS03, NED05, NICE06] | |
| Acute rhinitis | 2 | x | x | x | |
| Acute sinusitis | 13 | 9 (69.2)  [AUS03, CAN02, DEN02, NICE07, USA04, USA05, USA10, USA15, USA21] | 4 (30.8)  [AUS03, USA10, USA15, USA21] | 3 (23.1)  [AUS03, NED03, NICE07) | |
| Acute sore throat | 10 | 5 (50.)  [GER08, INT02, NICE10, USA21, BEL01] | 4 (40)  [AUS03, GER08, INT02, USA21] | 3 (30)  [AUS03, NED03, NICE10] | |
| Acute tonsillitis | 1 | x | x | x | |
| Conjunctivitis | 3 | x | x | 1 (33.3)  [NED04] | |
| Acute exacerbation of COPD^‡^ | 4 | x | 1 (25)  [AUS03] | 2 (50)  [AUS03, NICE 03] | |
| Croup Syndrome | 3 | 1 (33.3)  [NED01] | x | x | |
| Influenza | 4 | x | x | x | |
| URTI (unspecified) | 2 | 1(50)  [QAT01] | 1(50)  [QAT01] | x | |
| **Urinary system** | | | | | |
| Asymptomatic bacteriuria | 5 | 1(20)  [USA08] | x | x | |
| Recurrent UTI | 3 | x | 1(33.3)  [CAN04] | 1 (33.3)  [NICE09] | |
| Urinary tract infection | 18 | 7 (38.9)  [FIN06, GER04, NED08, NICE08, SCT03, CAN05] | 5 (27.8)  [FIN06, GER04, SCT03, USA20] | 2 (11.1)  [NED08, NICE08] | |
| **Skin and soft tissue system** | | | | | |
| Acne vulgaris | 5 | x | x | x | |
| Boils, carbuncles, abscesses | 3 | x | x | x | |
| Cellulitis and erysipelas | 5 | 1(20)  [NICE05] | 1 (20)  [NICE02] | x | |
| Impetigo | 6 | 1 (16.7)  [NICE05] | x | x | |
| Non-purulent SSTI | 1 | x | x | x | |

******Data reported as a percentage unless mentioned* ‘x’*indicates that no guideline reported antibiotic stewardship recommendation for the condition* † *Number of guidelines that reported each of the condition, ‡Chronic Obstructive Pulmonary Disease*

**Additional file H: Guidelines and examples of verbatim reporting of delayed prescribing**

| System | **Extended reporting of delayed prescribing** |  | **Basic reporting of delayed prescribing** |
| --- | --- | --- | --- |
| Respiratory | **Acute otitis media** | | |
|  | ***Examples*** |  |  |
|  | **DEN02:** Recommendations for antibiotic treatment depend on the age of the child and the severity of the symptoms. In other cases, you can usually afford to look at the time for 3 days without antibiotic treatment. One may consider using a “wait and see” prescription for patients with AOM. When prescribing a wait-and-see prescription, a time-limited prescription for an antibiotic is prescribed, but the patient is explained that the prescription should not be redeemed on the same day. | 3 days | **NED05:** The working group is of the opinion that in children with acute otitis media, provided they are not severe being ill and not belonging to a risk group, the benefits of starting an antibiotic immediately outweigh the drawbacks. A wait-and-see policy is therefore justified. |
|  | **Acute sinusitis** | | |
|  | **NICE07:** For people presenting with symptoms for around 10 days or more with no improvement: Consider no antibiotic prescription or a backup antibiotic prescription. Using the backup prescription if symptoms do not improve within 7 days or if they worsen rapidly or significantly at any time. | 7 days | **AUS03:** It is a good clinical practice to closely observe children with symptoms of AOM without middle ear effusion (OS) without prescribing antibiotics. Generally, a watchful waiting approach without initial antibiotic treatment is recommended in Denmark. |
|  |  |  |  |
| **Urinary** | **Urinary tract infection** | | |
|  | **NICE08:** However, acute, uncomplicated lower UTI in non-pregnant women can be self-limiting and for some women delaying antibiotic treatment with a backup prescription to see if symptoms will resolve without antibiotic treatment may be an option.  When a backup antibiotic prescription is given, advise about: using the backup prescription if symptoms do not start to improve within 48 hours or if they worsen at any time | 2 days | **SCT03:** In an era of increasing resistance to antibiotics and increasing awareness of the need for shared decision making in healthcare it is important for patients presenting with UTI symptoms to have the opportunity to discuss options with their healthcare professional to determine the best approach. Some patients may prefer to avoid taking antibiotics and prefer a ‘watch and wait’ approach if symptoms are not severe. |
| **Skin and Soft tissue** | **Impetigo** | | |
|  |  |  | **Example** |
|  | No guidelines reported delayed prescribing to an extended level |  | **NICE05:** This guideline also recommends that safety netting advice should be given to everyone who has an infection (regardless of whether or not they are prescribed or supplied with antimicrobials). |

*Extended: guidelines provided extensive details including the waiting period before redeeming the prescription.*

*Basic: guidelines recommended delayed prescribing but do not provide any additional information on the waiting period.*

**Additional file I:** **Examples of verbatim shared decision making recommendations in guidelines**

| **System** | ***SDM mentioned to an extended level**** | | ***SDM mentioned to the basic level***** |
| --- | --- | --- | --- |
| **Respiratory** | ***Examples***  ***Acute Bronchitis*** | | |
|  | **AUS 03:** Reassure the patient that acute bronchitis is a self-limiting condition, caused by a virus in over 90% of cases. The severity of symptoms can range from mild to severe; severe symptoms do not indicate a bacterial cause nor the need for antibiotic therapy. Ask about the patient or carer’s expectations for the management of acute bronchitis. Many patients have an expectation of treatment with antibiotics. Effective communication with the patient about the role of antibiotics in acute bronchitis is essential. The discussion should address possible misconceptions about the effectiveness of antibiotic therapy and the expectation of an antibiotic prescription. Box 2.22 provides a useful template for these discussions and outlines the approach to managing acute bronchitis with symptomatic therapy and patient education. A graphic to support shared decision-making discussions has been created by the Australian Commission on Safety and Quality in Health Care. | **JPN03:** Recommendations in the guidelines should not apply to all patients, given their individually different backgrounds and clinical conditions. Clinicians should make clinical decisions comprehensively, using evidence and recommendations, including the guidelines, the experience and specialty of each clinician, the preferences of patients and their parents/guardians, and their values. In the 2018 Guidelines, the balance of benefits and harms for the patients and patient preferences, in addition to the evidence quality, were emphasized and described as the reason for the recommendations | |
|  | ***Acute sinusitis*** | | |
|  | **USA10:** Clinicians deciding whether or not to treat ABRS with antibiotics should also solicit and consider patient preference and determine the relevance of existing evidence to their specific practice setting and patient population. Some patients may place great value on avoiding antibiotic therapy, whenever possible, but others may request initial antibiotics because they value the small but significant increase in clinical improvement they provide. Regardless of which initial strategy is used, clinicians should provide patients with clear information on management options, including symptomatic relief. Clinicians may also find it helpful to evaluate the patient’s pre-existing knowledge and attitudes about antibiotic therapy and ABRS, because they could affect treatment preference | | **USA21:** Consider prescribing a delayed or an immediate antibiotic based on the degree of illness, comorbidities, and after shared decision-making discussion with patients who meet criteria for ABRS. |
| **Urinary** | **Urinary tract infection** | | |
|  | **SCT03:** Patients and healthcare professionals may have different perspectives on healthcare processes and outcomes. The involvement of patients in guideline development is important to ensure that guidelines reflect their needs and concerns and address issues that matter to them. All women who are symptomatic of UTI should receive appropriate verbal and/or written self-care advice at every consultation regardless of diagnosis and treatment.  The decision to use an NSAID or antibiotic should be shared between patient and prescriber and risks and benefits should be fully discussed and considered. | | **FIN06:** Because the research evidence is limited and we do not know for which group of patients the delayed treatment is appropriate, a decision on a possible delay will be made in consultation with the patient. The decision takes into account the severity of the symptoms and the risks of complications |
| **Skin and soft tissue** | ***Cellulitis and erysipelas*** | | |
|  | **NICE02:** Involve the person in a shared decision by discussing and taking account of the severity and frequency of previous symptoms, the risk of developing complications, underlying conditions (such as oedema, diabetes, or venous insufficiency) and their management, the risk of resistance with long-term antibiotic use, the person's preference for antibiotic use. | | No guidelines made SDM recommendations at the basic level. |

***SDM= Shared Decision Making***

****Extended reporting:*** *provide extensive information outlining the process of SDM, making clear recommendations to clinicians on the approach to take in involving patients in deciding on the type of intervention.*

*******Basic reporting:*** *provided* *minimal information, such as that the clinician should consider patients' values and preferences.*
